# School educational models and child mental health among K-12 students: a scoping review

**DOI:** 10.1186/s13034-022-00469-8

**Published:** 2022-04-27

**Authors:** Ting Yu, Jian Xu, Yining Jiang, Hui Hua, Yulai Zhou, Xiangrong Guo

**Affiliations:** 1 The International Peace Maternity & Child Health Hospital, Shanghai Key Laboratory of Embryo Original Diseases, Shanghai Jiao Tong University School of Medicine, No. 910 Hengshan Road, Shanghai, 200030 China; 2 MOE-Shanghai Key Laboratory of Children’s Environmental Health, Department of Child and Adolescent Healthcare, Xinhua Hospital, Shanghai Jiao Tong University School of Medicine, Shanghai, 200092 China

**Keywords:** Student mental health, Curriculum, Homework, Physical activity, Interpersonal relationship, After-school activity

## Abstract

**Background:**

The promotion of mental health among children and adolescents is a public health imperative worldwide, and schools have been proposed as the primary and targeted settings for mental health promotion for students in grades K-12. This review sought to provide a comprehensive understanding of key factors involved in models of school education contributing to student mental health development, interrelationships among these factors and the cross-cultural differences across nations and societies.

**Methods:**

This scoping review followed the framework of Arksey and O’Malley and holistically reviewed the current evidence on the potential impacts of school-related factors or school-based interventions on student mental health in recent 5 years based on the PubMed, Web of Science, Embase and PsycExtra databases.

**Results/findings:**

After screening 558 full-texts, this review contained a total of 197 original articles on school education and student mental health. Based on the five key factors (including curriculum, homework and tests, physical activities, interpersonal relationships and after-school activities) identified in student mental development according to thematic analyses, a multi-component school educational model integrating academic, social and physical factors was proposed so as to conceptualize the five school-based dimensions for K-12 students to promote student mental health development.

**Conclusions:**

The lessons learned from previous studies indicate that developing multi-component school strategies to promote student mental health remains a major challenge. This review may help establish appropriate school educational models and call for a greater emphasis on advancement of student mental health in the K-12 school context among different nations or societies.

**Supplementary Information:**

The online version contains supplementary material available at 10.1186/s13034-022-00469-8.

## Introduction

In recent years, mental health conditions among children and adolescents have received considerable attention as a public health concern. Globally about 10–20% of children and adolescents experience mental health problems [1, 2], and mental health problems in early life may have the potential for long-term adverse consequences [3, 4]. In 2019, the World Health Organization has pointed out that childhood and adolescence are critical periods for the acquisition of socio-emotional capabilities and for prevention of mental health problems [5]. A comprehensive multi-level solution to child mental health problems needs to be put forward for the sake of a healthier lifestyle and environment for future generations.

The school is a unique resource to help children improve their mental health. A few generations ago, schools’ priority was to teach the traditional subjects, such as reading, writing, and arithmetic. However, children are now spending a large amount of time at school where they learn, play and socialize. For some students, schools have a positive influence on their mental health. While for others, schools can present as a considerable source of stress, worry, and unhappiness, and hinder academic achievement [2]. According to Greenberg et al., today’s schools need to teach beyond basic skills (such as reading, writing, and counting skills) and enhance students’ social-emotional competence, characters, health, and civic engagement [6]. Therefore, universal mental health promotion in school settings is recognized to be particularly effective in improving students’ emotional well-being [2, 7].

Research evidence over the last two decades has shown that schools can make a difference to students’ mental health [8]. Previous related systematic reviews or meta-analyses focused on the effects of a particular school-based intervention on child mental health [9, 10] and answered a specific question with available research, however, reviews covering different school-related factors or school-based interventions are still lacking. An appropriate model of school education requires the combination of different school-related factors (such as curriculum, homework, and physical activities) and therefore needs to focus on multiple primary outcomes. Thus, we consider that a scoping review may be more appropriate to help us synthesize the recent evidence than a systematic review or meta-analysis, as the wide coverage and the heterogeneous nature of related literature focusing on multiple primary outcomes are not amenable to a more precise systematic review or meta-analysis [11]. To the best of our knowledge, this review is among the first to provide a comprehensive overview of available evidence on the potential impacts of multiple school-related factors or school-based interventions on student mental health, and identify school-related risk/protective factors involved in the development of mental health problems among K-12 students, and therefore, to help develop a holistic model of K-12 education.

## Methods

### Design

A scoping review was systematically conducted following the methodological framework of Arksey and O'Malley [12]: defining the research question; identifying relevant studies; study selection; data extraction; and summarizing and reporting results. The protocol for this review was specified in advance and submitted for registration in the PROSPERO database (Reference number, CRD42019123126).

### Defining the research question (stage 1)

For this review, we sought to answer the following questions:

1. What is known from the existing literature on the potential impacts of school-related factors or school-based interventions on student mental health?
2. What are the interrelationships among these factors involved in the school educational process?
3. What are the cross-cultural differences in K-12 education process across nations and societies?

### Identifying relevant studies (stage 2)

The search was conducted in PubMed, Web of Science and Embase electronic databases, and the dates of the published articles included in the search were limited to the last 5 years until 23 March 2021. The PsycExtra database was also searched to identify relevant evidence in the grey literature [13]. In recent 5 years, mental disorders among children and adolescents have increased at an alarming rate [14, 15] and relevant policies calling for a greater role of schools in promoting student mental health have been issued in different countries [16–18], making educational settings at the forefront of the prevention initiative globally. Therefore, limiting research source published in the past 5 years was pre-defined since these publications reflected the newest discoveries, theories, processes, or practices. Search terms were selected based on the eligibility criteria and outcomes of interest were described as follows (Additional file 1: Table S1). The search strategy was peer-reviewed by the librarian of Shanghai Jiao Tong University School of Medicine.

### Study selection (stage 3)

T.Y. and Y.J. independently identified relevant articles by screening the titles, reviewing the abstracts and full-text articles. If any disagreement arises, the disagreement shall be resolved by discussion between the two reviewers and a third reviewer (J. X.).

Inclusion criteria were (1) according to the study designs: only randomized controlled trials (RCT)/quasi-RCT, longitudinal and cross-sectional studies; (2) according to the languages: articles only published in English or Chinese; (3) according to the ages of the subjects: preschoolers (3.5–5 years of age), children (6–11 years of age) and adolescents (12–18 years of age); and (4) according to the study topics: only articles examining the associations between factors involved in the school education and student mental health outcomes (psychological distress, such as depression, anxiety, stress, self-injury, suicide; and/or psychological well-being, such as self-esteem, self-concept, self-efficacy, optimism and happiness) in educational settings. Exclusion criteria: (1) Conference abstracts, case report/series, and descriptive articles were excluded due to overall quality and reliability. (2) Studies investigating problems potentially on a causal pathway to mental health disorders but without close associations with school education models (such as problems probably caused by family backgrounds) were excluded. (3) Studies using schools as the recruitment places but without school-related topics were also excluded.

### Data extraction (stage 4)

T.Y. and Y.J., and X.G., Y. Z., H.H. extracted data from the included studies using a pre-defined extraction sheet. Researchers extracted the following information from each eligible study: study background (name of the first author, publication year, and study location), sample characteristics (number of participants, ages of participants, and sex proportion), design [intervention (RCT or quasi-RCT), or observational (cross-sectional or longitudinal) study], and instruments used to assess exposures in school settings and mental health outcomes. For intervention studies (RCTs and quasi-RCTs), we also extracted weeks of intervention, descriptions of the program, duration and frequency. T.Y. reviewed all the data extraction sheets under the supervision of J. X.

### Summarizing and reporting the results (stage 5)

Results were summarized and reported using a narrative synthesis approach. Studies were sorted according to (a) factors/exposures associated with child and adolescent mental health in educational settings, and (b) components of school-based interventions to facilitate student mental health development. Key findings from the studies were then compared, contrasted and synthesized to illuminate themes which appeared across multiple investigations.

## Results

### Search results and characteristics of the included articles

**Fig. 1:**
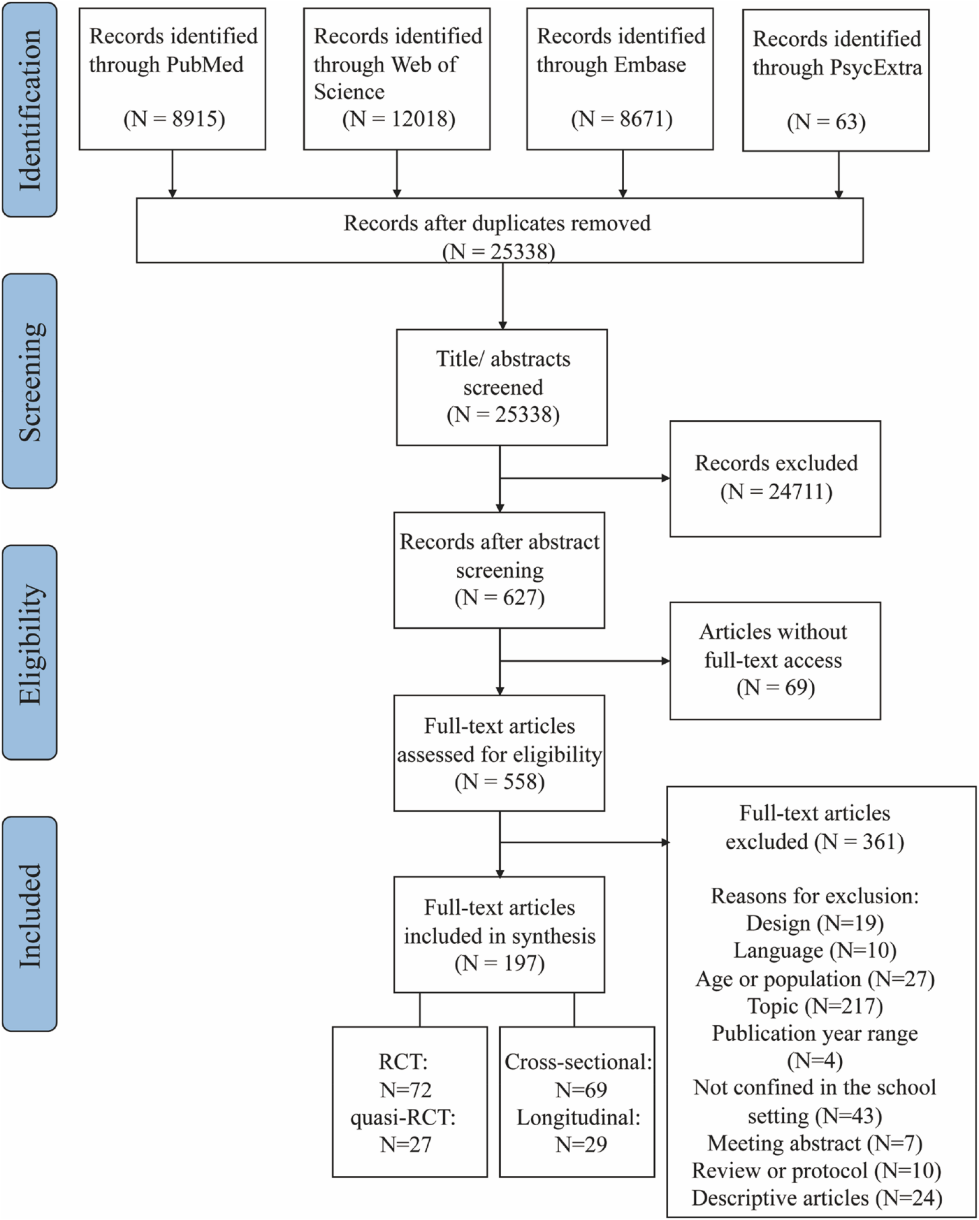
Study selection process

**Fig. 2:**
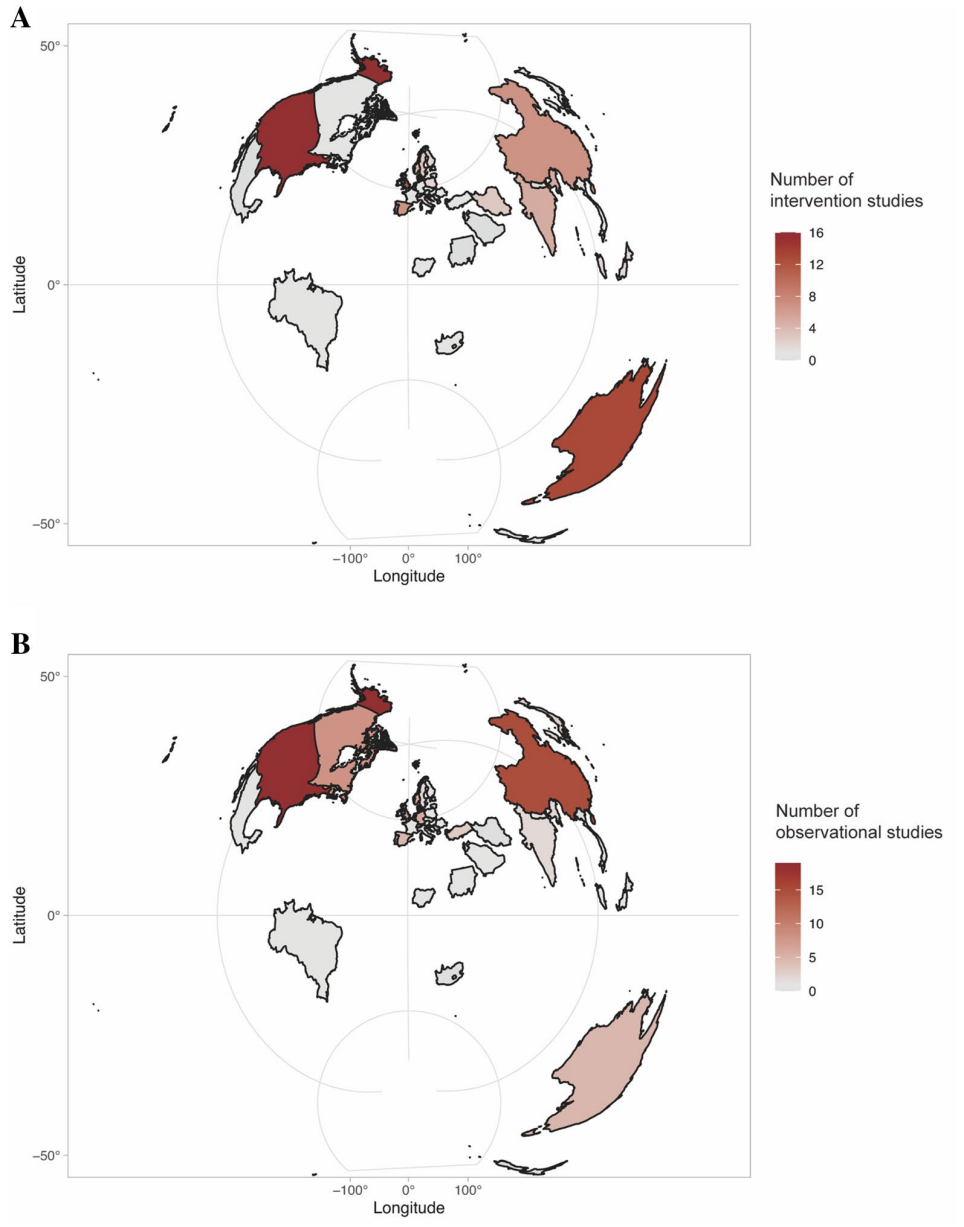
Geographical distribution of included studies: **A** intervention studies; **B** observational studies

The search yielded 25,338 citations, from which 558 were screened in full-text. Finally, a total of 197 original articles were included in this scoping review: 72 RCTs (including individually randomized and cluster-randomized trials), 27 quasi-RCTs, 29 longitudinal studies and 69 cross-sectional studies (Fig. 1 for details). Based on thematic analyses, the included studies were analyzed and thematically grouped into five overarching categories based on the common themes in the types of intervention programs or exposures in the school context: curriculum, homework and tests, interpersonal relationships, physical activity and after-school activities. Table 1 provided a numerical summary of the characteristics of the included articles. The 197 articles included data from 46 countries in total, covering 24 European countries, 13 Asian countries, 4 American countries, 3 African countries, and 2 Oceanian countries. Most intervention studies were conducted in the United States of America (n = 16), followed by Australia (n = 11) and the United Kingdom (n = 11). Most observational studies were conducted in the United States of America (n = 19), followed by China (n = 15) and Canada (n = 8). Figure 2 illustrated the geographical distribution of the included studies. Further detailed descriptions of the intervention studies or observational studies were provided in Additional file 1: Tables S2 and S3, respectively.

**Table 1:** Summary of the included articles

Characteristics of the reviewed articles	Number of articles	% of articles
Category		
Curriculum	68	34.5
Homework and tests	17	8.6
Interpersonal relationships	60	30.5
Physical activity	25	12.7
After-school activity	23	11.7
Multi-component	4	2.0
Study design		
RCTs	72	36.6
quasi-RCTs	27	13.7
Longitudinal	29	14.7
Cross-sectional	69	35.0
Age of participants		
Preschoolers (3.5–5 years of age)	5	2.5
Children (6–12 years of age)	50	25.4
Adolescents (12–18 years of age)	142	72.1
Sample size		
Small (< 100 participants)	21	10.7
Medium (100–300 participants)	38	19.3
Large (> 300 participants)	138	70.0
Sex ratios of participants		
Males > 60%	10	5.1
Females > 60%	26	13.2
Fairly distributed (50–60% males or females)	151	76.6
Not shown	10	5.1

### Curriculum

The association between school curriculum and student mental health was investigated in four cross-sectional studies. Mathematics performance was found to be adversely associated with levels of anxiety or negative emotional responses among primary school students [19]. However, in middle schools, difficulties and stressors students may encounter in learning academic lessons (such as difficulties/stressors in taking notes and understanding teachers’ instructions) could contribute to lowered self-esteem [20] and increased suicidal ideation or attempts [21]. Innovative integration of different courses instead of the traditional approach of teaching biology, chemistry, and physics separately, could improve students’ self-concept [22].

**Fig. 3:**
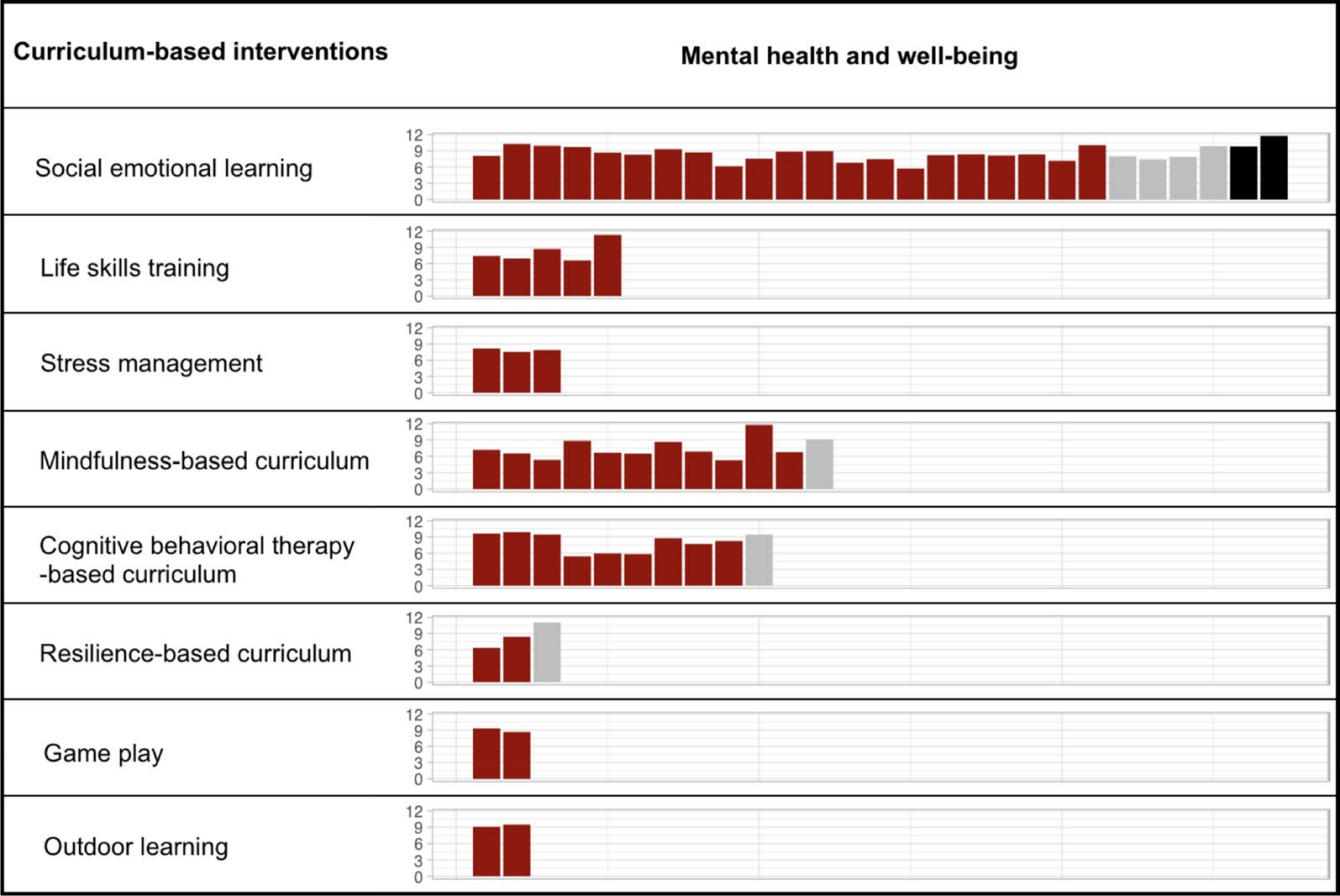
Harvest plots for overview of curriculum-based intervention studies, grouped by different types of curriculum-based interventions. The height of the bars corresponded to the sample sizes on a logarithmic scale of each study. Red bars represented positive effects of interventions on student mental health outcomes, grey bars represented non-significant effects on student mental health outcomes, and black bars represented negative effects on student mental health outcomes

To promote student mental health, 64 intervention studies were involved in innovative curricula integrating different types of competencies, including social emotional learning (SEL), mindfulness-intervention, cognitive behavioral therapy (CBT)-based curriculum, life skills training, stress management curriculum, and so on (Fig. 3). Curricula focusing on SEL put an emphasis on the development of child social-emotional skills such as managing emotions, coping skills and empathy [23], and showed positive effects on depression, anxiety, stress, negative affect and emotional problems [23–37], especially in children with psychological symptoms [24] and girls [23, 27], as well as increased prosocial behaviors [38], self-esteem [39–42] and positive affect [43]. However, four programs reported non-significant effects of SEL on student mental health outcomes [44–47], while two programs demonstrated increased levels of anxiety [48] and a reduction of subjective well-being [49] at post-intervention. Mindfulness-based curriculum showed its potential to endorse positive outcomes for youth including reduced emotional problems and negative affect [50–56] as well as increased well-being and positive emotions [51, 52, 57–60], especially among high-risk children with emotional problems or perceived stress before interventions [50, 53]. However, non-significant effects were also reported in an Australian study in secondary schools [61]. Curricula based on CBT targeted children at risk or with early symptoms of mental illness [62–67], or all students regardless of symptom levels as a universal program [68–70], and could impose a positive effect on self-esteem, well-being, distress, stress and suicidality. However, a universal CBT trial in Swedish primary schools found no evidence of long-term effects of such program on anxiety prevention [71]. Five intervention studies based on life-skill-training were found to be effective in promoting self-efficacy [72, 73], self-esteem [73, 74], and reducing depression/anxiety-like symptoms [72, 75, 76]. Courses covering stress management skills have also been reported to improve life satisfaction, increase happiness and decrease anxiety levels among students in developing countries [77–79]. In practice, innovative teaching forms such as the game play [67, 80, 81] and outdoor learning [82, 83] embedded in the traditional classes could help address the mental health and social participation concerns for children and youth. Limited evidence supported the mental health benefits of resilience-based curricula [84–86], which deserve further studies.

Large cluster-randomized trials utilizing multi-component whole-school interventions which involves various aspects of school life (curriculum, interpersonal relationships, activities), such as the Strengthening Evidence base on scHool-based intErventions for pRomoting adolescent health (SEHER) program in India and the Together at School program in Finland, have been proved to be beneficial for prevention from depression [87–89] and psychological problems [90].

### Homework and tests

#### Homework

The association between homework and psychological ill-being outcomes was investigated in four cross-sectional studies and one longitudinal study. Incomplete homework and longer homework durations were associated with a higher risk of anxiety symptoms [91, 92], negative emotions [93–95] and even psychological distress in adulthood [96].

#### Tests

Innumerable exams during the educational process starting from primary schools may lead to increased anxiety and depression levels [97, 98], particularly among senior students preparing for college entrance examinations [99]. Students with higher test scores had a lower probability to have emotional and behavioral problems [100], in comparison with students who failed examinations [93, 101]. Depression and test anxiety were found to be highly correlated [102]. In terms of psychological well-being outcomes, findings were consistent in the negative associations between student test anxiety and self-esteem/life-satisfaction levels [103, 104]. Regarding intervention studies, adolescent students at a high risk of test anxiety benefited from CBT or attention training by strengthening sense of control and meta-cognitive beliefs [105, 106]. However, more knowledge about the criteria for an upcoming test was not related to anxiety levels during lessons [107].

### Interpersonal relationships

School-based interpersonal (student–student or student–teacher) relationships are also important to student mental health. Low support from schoolmates/teachers and negative interpersonal events were reported to be associated with psychosomatic health complaints [108–113]. In contrast, positive interpersonal relationships in schools could promote emotional well-being [114–117] and reduce depressive symptoms in students [118–120].

#### Student–teacher relationships

Negative teaching behaviors were associated with negative affect [121, 122] and low self-efficacy [123] among primary and high school students. Student–teacher conflicts at the beginning of the school year were associated with higher anxiety levels in students at the end of the year, and high-achieving girls were most susceptible to such negative associations [124]. Higher levels of perceived teachers’ support were correlated with decreased risks of depression [125], mental health problems [126] as well as increased positive affect [127, 128] and improved mental well-being [129, 130]. Better student–teacher relationships were positively associated with self-esteem/efficacy [131], while negatively associated with the risks of adolescents’ externalizing behaviors [132] among secondary school students. Longitudinal studies demonstrated that high intimacy levels between students and teachers were correlated with reduced emotional symptoms [133] and increased life-satisfaction among students [134]. In addition, more respect to teachers in 10th grade students was associated with higher self-efficacy and lower stress levels 1 year later [135].

A growing body of research focused on the issue of how to increase positive interactions between teachers and students in teaching practices. Actually, interventions on improving teaching skills to promote a positive classroom atmosphere could potentially benefit children, especially those experiencing a moderate to high level of risks of mental health problems [136, 137].

#### Student–student relationships

Findings were consistent in considering the positive peer relationship as a protective factor against internalizing and externalizing behaviors [138–142], depression [143–145], anxiety [146], self-harm [147] and suicide [148], and as a favorable factor for positive affect [149, 150], increased happiness [151], self-efficacy [152], optimism [153, 154] and mental well-being [155]. In contrast, peer-hassles, friendlessness, negative peer-beliefs, peer-conflicts/isolation and peer-rejection, have been identified in the development of psychological distress among students [141, 143, 149, 156–165].

As schools and classrooms are common settings to build peer relationships, student social skills to enhance the student–student relationship can be incorporated into school education. Training of interpersonal skills among secondary school students with depressive symptoms appeared to be effective in decreasing adolescent internalizing and externalizing symptoms [166]. In addition, recent studies also identified the effectiveness of small-group learning activities in the cognitive development and mental health promotion among students [87–90, 167].

### Physical activity in school

Moderate-to-high-intensity physical activity during school days has been confirmed to benefit children and adolescents in relation to various psychosocial outcomes, such as reduced symptoms of depression [168], emotional problems [169] and mental distress [170] as well as improved self-efficacy [171] and mental well-being [172, 173]. In addition, participation in physical education (PE) at least twice a week was significantly associated with a lower likelihood of suicidal ideation and stress [174].

A variety of school‐based physical activity interventions or lessons have been proposed in previous studies to promote physical activity levels and psychosocial fitness in students, including integrating physical activities into classroom settings [175–178], assigning physical activity homework [178], physically-active academic lessons [179, 180] as well as an obligation of ensuring the participation of various kinds of sports (such as aerobic exercises, resistance exercises, yoga) in PE lessons [181–192]. Although the effectiveness of these proposed physical activity interventions was not consistent, physical education is suggested to implement sustainably as other academic courses with special attention.

### After-school activities

Several cross-sectional studies have synthesized evidence on the positive effects of leisure-time physical activity against student depression, anxiety, stress, and psychological distress [193–199]. Extracurricular sport participation (such as sports, dance, and martial arts) could foster perceived self-efficacy, self-esteem, improve mental health status [200–203], and reduce emotional problems [204] and depressive symptoms [205]. Participation in team sports was more strongly related to beneficial mental health outcomes than individual sports, especially in high school girls [199]. Other forms of organized activities, such as youth organizations and arts, have also been demonstrated to benefit self-esteem [201], self-worth [206], satisfaction with life and optimism [207, 208].

However, different types of after-school activities may result in different impacts on student mental health. Previous studies demonstrated that students participating in after-school programs of yoga or sports had better well-being and self-efficacy [209], and decreased levels of anxiety [210] and negative mood [211], while another study showed that the after-school yoga program induced no significant changes in levels of depression, anxiety and stress among students [212]. Inconsistent findings on the effects of participation in art activities on student mental health were also reported [213, 214]. Another study also highlighted the benefits of after-school clubs, demonstrating an improvement in socio-emotional competencies and emotional status, and sustained effects at 12-month follow-up [215].

## Discussion

**Fig. 4:**
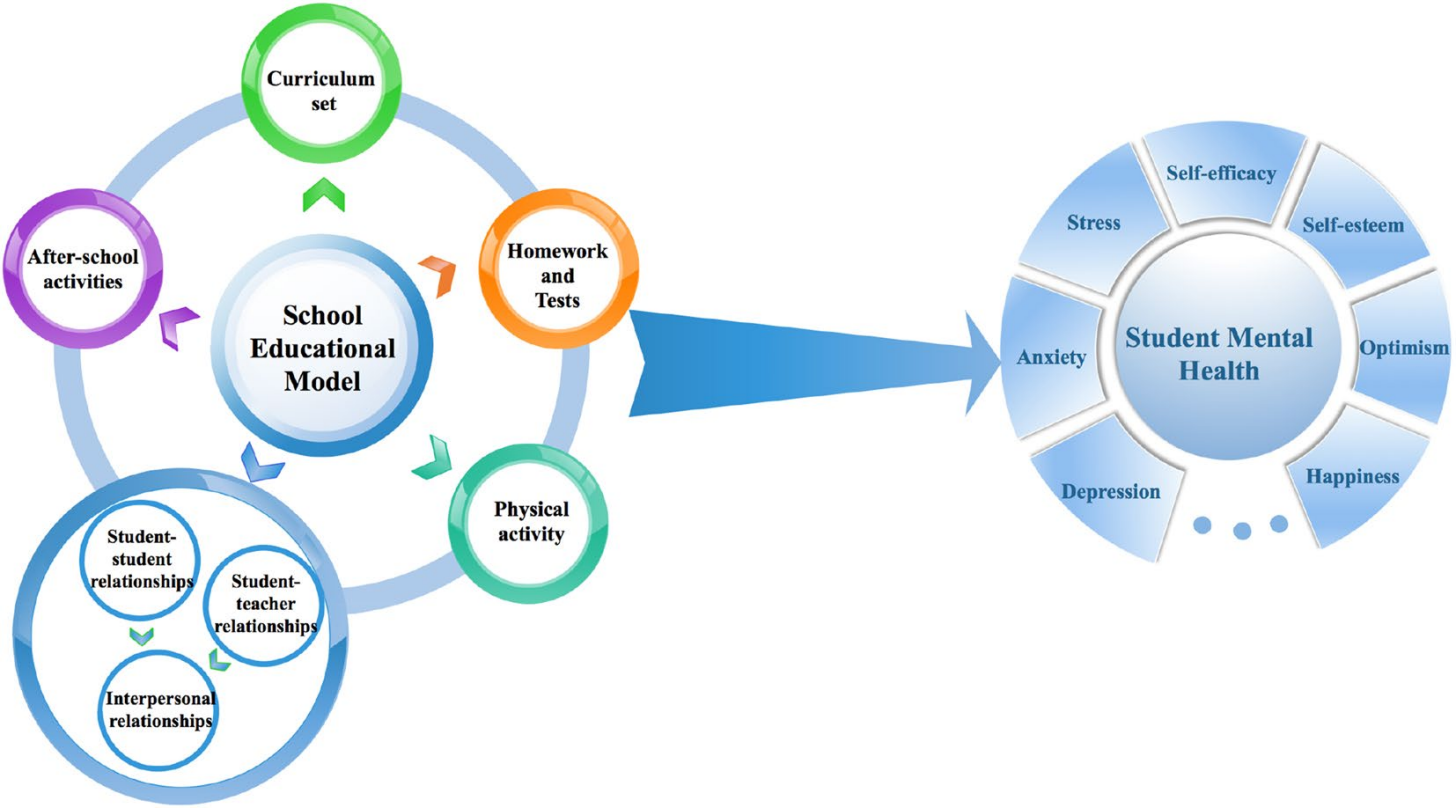
The multi-component school educational model is proposed to conceptualize the five school-based dimensions (including curriculum set, homework and tests, physical activity, interpersonal relationships and after-school activities) for K-12 students to promote student mental health

Based on the potential importance of the five school-based factors identified in student mental development, a multi-component school educational model is therefore proposed to conceptualize the five school-based dimensions (including curriculum, homework and tests, interpersonal relationships, physical activity, and after-school activities) for K-12 students to promote their mental health (Fig. 4). The interrelationships among the five dimensions and cross-cultural comparisons are further discussed as follows in a holistic way.

### Comprehensive understanding of K-12 school educational models: the reciprocal relationships among factors

Students’ experiences in the school educational context are dynamic processes which englobe a variety of educational elements (such as curriculum, homework, tests) and social elements (such as interpersonal relationships and social activities in schools). Based on the educational model proposed in this review, these educational/social elements are closely related and interact with each other, which play an important role in students’ psychosocial development.

Being aware of this, initiatives aimed to improve student social and emotional competencies may certainly impact student psychological well-being, at least in part, in a way of developing supportive relationships between teachers-students or between peers [35, 89]. On the other hand, the enhancement of interpersonal relationships at school could serve as a potent source of motivation for student academic progress so as to further promote psychological well-being [131, 132]. In addition, school education reforms intended to provide pupils with more varied teaching and learning practices to promote supportive interpersonal relationships between students and teachers or between peers, such as education programs outside the classroom [82], cooperative learning [167] and adaptive classroom management [136, 137], have also been advocated among nations recently.

Our findings also suggested that participation in non-academic activities was an important component of positive youth development. Actually, these school-based activities in different contexts also require teacher–student interactions or peer interactions. Social aspects of physical activities have been proposed to strengthen relationship-building and other interpersonal skills that may additionally protect students against the development of mental health problems [130, 203]. Among various types of sports, team sports seemed to be associated with more beneficial outcomes compared with individual sports due to the social aspect of being part of a team [194, 199]. Participation in music, student council, and other clubs/organizations may also provide students with frequent connections with peers, and opportunities to build relationships with others that share similar interests [201]. Further, frequent and supportive interactions with teachers and peers in sports and clubs may promote student positive views of the self and encourage their health-promoting behaviors (such as physical activities).

However, due to increasing academic pressure, children have to spend a large amount of time on academic studies, and inevitably displace time on sleep, leisure, exercises/sports, and extracurricular activities [92]. Although the right amount of homework may improve school achievements [216] and higher test scores may help prevent students from mental distress [100–102], over-emphasis on academic achivements may lead to elevated stress levels and poor health outcomes ultimately. The anxiety specifically related to academic achievement and test-taking at school was frequently reported among students who felt pressured and overwhelmed by the continuous evaluation of their academic performance [98, 103, 104]. In such high-pressure academic environments, strategies to alleviate the levels of stress among students should be incorporated into intervention efforts, such as stress management skill training [77–79], CBT-based curriculum [62, 64, 66, 105], and attention training [106]. Therefore, school supportive policies that allow students continued access to various non-academic activities as well as improve their social aspect of participation may be one fruitful avenue to promote student well-being.

### Cross-cultural differences in K-12 educational models among different nations and societies

As we reviewed above, heavy academic burden exists as an important school-related stressor for students [91, 92, 94–96], probably due to excessive examinations [97–99] and unsatisfactory academic performance [100–102]. Actually, extrinsic cultural factors significantly impact upon student academic burden. In most countries, college admission policies affect the entire ecological system of K-12 education, because success in life or careers is determined by examination performance to a large extent [217]. The impacts of heavy academic burden may be greatest in Asian cultures where more after-school time of students is spent on homework, exam preparations, and extracurricular classes for academic improvement (such as in Korea, Japan, China and Singapore) [92, 95, 218]. As a consequence, the high proportion of adolescents fall in the “academic burnout group” in Asian countries [219], which highlights the need to take further measures to combat the issue. As an issue of concern, the “double reduction” policy has been implemented nationwide in China since 2021, being aimed to relieve students of excessive study burden, and the effects of the policy are anticipated but remain unknown up to now.

Other factors such as school curriculum and extra-curricular commitments, vary among societies and nations and may explain the cross-cultural differences in educational models [220]. For example, in Finland, the primary science subject is as important as mathematics or reading, while Chinese schools often lack time to arrange a sufficient number of science courses [221], which could be explained by different educational traditions of the two countries. In addition, approximately 75% of high schools in Korea failed to implement national curriculum guidelines for physical education (150 min/week), instead replacing that time with self-guided study to prepare for university admission exams [174]. In terms of the arrangement of the after-school time, Asian students spend most of their after-school time on private tutoring or doing homework [222], 2–3 times longer than the time spent by adolescents in most western countries/cities [92]. However, according to our analyses and summaries, most intervention studies targeting the improvement of mental health of students by school education were conducted in western countries (Fig. 2), suggesting that special attention needs to be paid to the students’ mental health issue on campus, especially in countries where students have heavy study-loads. Merits of the different educational traditions also need to be considered in the designs of educational models among different countries.

### Strengths and limitations

This study focuses on an interdisciplinary topic covering the fields of developmental behavioral pediatrics and education, and the establishment of appropriate school educational models is teamwork involving multiple disciplines including pediatrics, prevention, education, services and policy. Although there are lots of studies focusing on a particular factor in school educational processes to promote student mental health, comprehensive analysis/understanding on multi-component educational model is lacking, which is important and urgently needed for the development of multi-dimensional educational models/strategies. Therefore, we included a wide range of related studies, summarized a comprehensive understanding of the evidence base, and discussed the interrelationships among the components/factors of school educational models and the cross-cultural gaps in K-12 education across different societies, which may have significant implications for future policy-making.

Some limitations also exist and are worth noting. First, this review used the method of the scoping review which adopted a descriptive approach, rather than the meta-analysis or systematic review which provided a rigorous method of synthesizing the literature. Under the subject (appropriate school education model among K-12 students) of this scoping review, multiple related topics (including curriculum, homework and tests, physical activities, interpersonal relationships and after-school activities) were included rather than one specific topic. Therefore, we consider that the method of the scoping-review is appropriate, given that the aim of this review is to chart or map the available literature on a given subject rather than answering a specific question by providing effect sizes across multiple studies. Second, we limited the study search within recent 5 years. Although we consider that the fields involved in this scoping review change quickly with the acquisition of new knowledge/information in recent 5 years, limiting the literature search within recent 5 years may make us miss some related but relatively old literature. Third, we only included studies disseminated in English or Chinese, which may limit the generalizability of our results to other non-English/Chinese speaking countries.

## Conclusion

This scoping review has revealed that the K-12 schools are unique settings where almost all the children and adolescents can be reached, and through which existing educational components (such as curriculum, homework and tests, physical activities, interpersonal relationships and after-school activities) can be leveraged and integrated to form a holistic model of school education, and therefore to promote student mental health. In future, the school may be considered as an ideal setting to implement school-based mental health interventions. Our review suggests the need of comprehensive multi-component educational model, which involves academic, social and physical factors, to be established to improve student academic achievement and simultaneously maintain their mental health.

However, questions still remain as to what is optimal integration of various educational components to form the best model of school education, and how to promote the wide application of the appropriate school educational model. Individual differences among students/schools and cross-cultural differences may need to be considered in the model design process.

## Supplementary Information

**Additional file 1: Table S1.** Search strategies used for each database. **Table S2**. Summaries of intervention studies (randomized/quasi-randomized controlled trials) investigating the effects of school-based interventions on child mental health (n = 99). **Table S3.** Summaries of observational research on relationships between school-related factors and student mental health outcomes (n = 98).

## Data Availability

The data analysed in this review are available from the corresponding author upon request.

## Abbreviations

CBT: Cognitive behavioral therapy
PE: Physical education
RCT: Randomized controlled trials
SEL: Social emotional learning
