# Supplementary material for: School educational models and child mental health among K-12 students: a scoping review
Source: Child Adolesc Psychiatry Ment Health. 2022 Apr 27;16:32. doi: 10.1186/s13034-022-00469-8 (PMC9047301; doi:10.1186/s13034-022-00469-8)
Supplement: Supplementary file 1 — Additional file 1: Table S1. Search strategies used for each database. Table S2. Summaries of intervention studies (randomized/quasi-randomized controlled trials) investigating the effects of school-based interventions on child mental health (n = 99). Table S3. Summaries of observational research on relationships between school-related factors and student mental health outcomes (n = 98). [file 13034_2022_469_MOESM1_ESM.docx]

**Table S1: Search strategies used for each database**

| **Database** | **Search strategies** |
| --- | --- |
| **PubMed** | ((((((((((Child[MeSH Terms])) OR (Child, Preschool[MeSH Terms])) OR (Adolescent[MeSH Terms])) OR (Puberty[MeSH Terms])) OR (boys[MeSH Terms])) OR (girls[MeSH Terms])) OR (childhood) OR (school-age)) AND ((((((((((Mental Health[MeSH Terms]) OR (Self Concept[MeSH Terms])) OR (Self Efficacy[MeSH Terms])) OR (Happiness[MeSH Terms])) OR (Affect[MeSH Terms])) OR (Optimism[MeSH Terms])) OR (Anxiety[MeSH Terms])) OR (Depression[MeSH Terms])) OR (Pessimism[MeSH Terms])) OR (Stress, Psychological[MeSH Terms]))) AND (((((((((preschool[Title/Abstract]) OR (kindergarten[Title/Abstract])) OR (school*[Title/Abstract])) OR (education*[Title/Abstract])) OR (classroom*[Title/Abstract])) OR (curricul*[Title/Abstract])) OR (teacher[Title/Abstract])) OR (student*[Title/Abstract])) OR (peer*[Title/Abstract]))  Limit：((y_5[Filter]) AND (humans[Filter]) AND (chinese[Filter] OR english[Filter]) AND (preschoolchild[Filter] OR child[Filter] OR adolescent[Filter])) |
| **Web of Science** | 1.(Child*) OR (Preschool child) OR (Adolescent) OR (Teen) OR (youth) OR (boys) OR (girls) OR (school-age) OR (childhood)  2.(Mental Health) OR ("Self Concept") OR ("Self Efficacy") OR ("Anxiety") OR ("Depression") OR ("Pessimism") OR ("Stress") OR ("Optimism")  3.title:(school*) OR title: (education*) OR title: (classroom*) OR title: (curricul*) OR title: (teacher) OR title: (student*) OR title: (peer*)  4. #1 AND #2 AND 3  Limit: Publication date: recent 5 years; Ages: Children and adolescents: 2–18 years. |
| **Embase** | 1.‘child’/exp OR ‘preschool’/exp OR ‘adolescent’/exp OR ‘puberty’/exp OR boys OR girls OR ‘Childhood’/exp OR ‘school age’/exp  2.‘mental health’/exp OR ‘self concept’/exp OR ‘self efficacy’/exp OR ‘happiness’/exp OR ‘affect’/exp OR ‘optimism’/exp OR ‘anxiety’/exp OR ‘depression’/exp OR ‘pessimism’/exp OR ‘stress’/exp  3.school*:ti,ab,kw OR education*:ti,ab,kw OR classroom*:ti,ab,kw OR curricul*:ti,ab,kw OR teacher:ti,ab,kw OR student*:ti,ab,kw OR peer*:ti,ab,kw  4.#1 AND #2 AND #3 AND ([chinese]/lim OR [english]/lim) AND ([preschool]/lim OR [school]/lim OR [adolescent]/lim) AND [humans]/lim AND [embase]/lim AND [2017-2021]/py |
| **PsycExtra (Grey literature)** | ((child) OR (preschool child) OR (adolescent) OR (teen) OR (youth) OR (boys) OR (girls) OR (school-age) OR (childhood)) AND ((Mental Health) OR (Self Concept) OR (Self Efficacy) OR (Anxiety) OR (Depression) OR (Pessimism) OR (Stress) OR (Optimism)) AND ((AB school*) OR (AB education*) OR (AB classroom*) OR (AB curricul*) OR (AB teacher) OR (AB student*) OR (AB peer*))  Limiters-English; Published Date: 20160301-20210331; Age groups: Preschool Age (2-5yrs), School Age (6-12 yrs), Adolescence (13-17 yrs); Population group: Human |

**Table S2: Summaries of intervention studies (randomized/ quasi-randomized controlled trials) investigating the effects of school-based interventions on child mental health (n = 99).**

| Category | Program content | Author, year, country | Intervention/sample number (age range; % girls)  Design; population | Descriptions of intervention; duration; frequency | Mental health indicators; Instruments | Main findings |
| --- | --- | --- | --- | --- | --- | --- |
| Curriculum | Social emotional learning (SEL) | Burckhardt R et al., 2016 [1], Australia | 139/267 (15-18 years old; 39.3% girls)  cluster-RCT; high schools | SEL VS. usual ‘Pastoral Care’ classes; 16 30-min sessions during 3 months; twice a week | Anxiety, depression and stress; Depression Anxiety Stress Scale – Short form (DASS-21)  Subjective wellbeing; Flourishing scale (FS) | The interventions showed positive effects on student mental health outcomes. |
|  |  | Coelho VA et al., 2016 [2] , Portugal | 970/1237 (mean age=11.22 years old; 46.7% girls)  quasi-RCT; primary schools (4th grade) | SEL VS. control; 13 one-hour sessions; weekly | Self-esteem; The Global Self-Esteem scale of the Self- Description Questionnaire I |  |
|  |  | Coelho VA et al., 2017 [3], Portugal | 355 (curriculum format) & 309 (pre-package format)/982 (mean age=11.22 years old; 46.8% girls)  quasi-RCT; low middle schools (5-6th grade) | SEL in curriculum format VS. pre-package format VS. control; 13 one-hour sessions; weekly | Self-esteem; The Global Self-Esteem scale of the Self- Description Questionnaire I |  |
|  |  | Coelho VA et al., 2018 [4], Portugal | 319 (after-school curriculum) & 272 (with-school curriculum)/837 (mean age=12.70 years old; 47.6% girls)  quasi-RCT; middle schools (7-9th grade) | SEL in school VS. after school VS. control; 13 one-hour sessions; weekly | Self-esteem; The Global Self-Esteem scale of the Self- Description Questionnaire II |  |
|  |  | Hoang-Minh D et al., 2017 [5], Vietnam | 202/404 (Mean age = 8.71 years old; 49% girls)  cluster-RCT; primary schools | SEL VS. control; 1 academic year (45 min); once to twice per week | Internalizing mental health problems and externalizing problems; Student Behavior Questionnaire |  |
|  |  | Shoshani A et al., 2017 [6], Israel | 160/315 (3–6.5 years old; 48.6% girls)  cluster-RCT; kindergartens | SEL VS. control; 32 weeks; 5 activities per week | Positive and negative affect (child and parent report); The Shortened Positive and Negative Affect Scale for Children (PANAS-C) |  |
|  |  | Roberts CM et al., 2018 [7], Australia | 630 (SEL with teacher training) & 863 (SEL with teacher training plus coaching)/2288 (10-11 years old; 48.9% girls)  cluster-RCT; primary schools | SEL with teacher training VS. SEL with teacher training plus coaching VS. control; 2 years (1 hour); 20 lessons in two years | Mental health problems; The student version of the Strengths and Difficulties Questionnaire (SDQ-S) Depressive and anxiety disorders; The Diagnostic Interview for Children and Adolescents IV (DICA-IV) |  |
|  |  | Garaigordobil M et al., 2018 [8], Spain | 218/420 (7-10 years old; 46.7% girls)  Cluster-RCT; primary schools | SEL VS. cooperative play curriculum; 18 weeks; weekly | Depression; Child Depression Scale, Children’s Depression Scale-Teacher Internalizing problems, externalizing problems； Screening for Children’s Emotional and Behavioral Problems |  |
|  |  | Flynn D et al.,2018 [9], Ireland | 26/71(15-16 years old; 100% girls)  quasi-RCT; post-primary schools | SEL VS. control; 22 weeks; not shown | Emotional wellbeing; The Emotional Symptom Index |  |
|  |  | Young JF et al., 2018 [10], USA | 95/186 (Mean age = 14.01 years old; 66.7% girls)  RCT; middle and high school students with elevated depressive symptoms | SEL VS. group counseling; not shown; not shown | Depression; Center for Epidemiologic Studies–Depression Scale (CES-D) |  |
|  |  | Ab Ghaffar SF et al., 2019 [11], Malaysia | 193/461 (10-11 years old; 56.0% girls)  cluster-RCT; primary schools | SEL VS. control; 4 weeks (60 min); weekly | Anxiety; The Revised Child Anxiety and Depression Scale (RCADS 25) Self-esteem; The Rosenberg Self-Esteem Scale |  |
|  |  | Dowling K et al., 2019 [12], Ireland | 246/497 (15-18 years old; 51.3% girls)  cluster-RCT; disadvantaged post-primary schools | SEL VS. control; 13 sessions; weekly | Mental health; The Depression Anxiety Stress Scale  Mental wellbeing; The Warwick Edinburgh Mental Wellbeing Scale (WEMWBS) |  |
|  |  | yer RB et al., 2019 [13], USA | 74/112 (13-15 years old; 51.8% girls)  quasi-RCT; middle schools | SEL VS. optional curriculum; 13 weeks (45 min); 5 days a week | Stress; The 10- item Perceived Stress Scale (PSS)  Mental well-being; the Warwick-Edinburgh Mental Well-being Scale (WEMWBS) |  |
|  |  | Zhao Y et al., 2019 [14], China | 84/173 (Mean age = 13.54 years old; 53.2% girls)  cluster-RCT; middle schools | SEL VS. moral education class; 10 weeks (45 min); weekly | Depression; The five items that assess symptoms of depression in the patient-reported outcome measurement information system (PROMIS) pediatric eight-item short forms |  |
|  |  | Taghvaienia A et al., 2020 [15], Iran | 27/53 (15-18 years old; 100% girls)  RCT; high school students with mild-moderate depression | SEL VS. control; 8 weeks (2 hours); weekly | Depression; The Beck Depression Inventory (BDI-II)） |  |
|  |  | Johnstone KM et al., 2020 [16], Australia | 185 (Emotion Regulation) & 85 (Behavioral Activation)/295 (8-13 years  old; 52.5% girls)  cluster-RCT; primary schools | SEL VS. control; eight 50‐min sessions; weekly | Worry; Penn State Worry Questionnaire—Child version (PSWQ‐C) |  |
|  |  | Bono G et al., 2020 [17], USA | 152/327 (13-18 years old; 57% girls)  quasi-RCT; high schools | SEL VS. control; 6 weeks; not shown | Positive and negative affect; The Positive and Negative Affect Schedule for Children  Anxiety Symptoms; The Spence Children’s Anxiety Scale |  |
|  |  | Maalouf FT et al., 2020 [18], USA | 145/280 (11-13 years old; 52.1% girls)  cluster-RCT; middle schools | SEL VS. control;10 50-min sessions; weekly | Psychological symptoms ; The Scale for Childhood Anxiety and Related Disorders (SCARED), Mood and Feelings Questionnaire (MFQ), and Strengths and Difficulties Questionnaire (SDQ) |  |
|  |  | Humphrey N et al., 2020 [19], UK | 160/326 (9-11 years old; not shown)  cluster-RCT; primary school students with emerging mental health difficulties | SEL VS. control;10 sessions; weekly | Emotional symptoms; ten items, sample item: “I worry a lot”; response format: 0 = never, 1 = sometimes, 2 = always)  Self-esteem; three items, sample item: “I can do most things if I try”; response format: 1 = never to 5 = always |  |
|  |  | Sánchez-Sansegundo M et al., 2020 [20], Spain | 68/142 (13–17 years old; 54.5% girls)  cluster-RCT; high schools | SEL VS. control; 6 months (2 hour); 2 sessions per month | Self-Esteem; The Rosenberg Self-Esteem Scale (RSES) |  |
|  |  | Zhu X et al., 2020 [21], China | 539/1044 (Mean age =13 years old; 74.6% girls)  cluster-RCT; junior secondary schools | SEL VS. regular class activities such as self-study or class meeting; 32 weeks (45 min); once every two weeks | Depression; The 20-item “Center for Epidemiologic Studies Depression Scale” (CES-D) |  |
|  |  | Tomyn JD et al., 2016 [22], Australia | 164/252 (13 to 17 years old; 40% girls)  cluster-RCT; secondary schools | SEL VS. regular health and wellbeing classes; six 50-min lessons; weekly | Depression; The 13-item Short Mood and Feelings Questionnaire (SMFQ) | The interventions showed no significant effects on student mental health outcomes. |
|  |  | Schanen JG et al., 2018 [23], USA | 81/164 (7-8^th^ grade ; data not shown)  quasi-RCT; middle schools | SEL VS. non-participating controls; 14 sessions during 1 year; not shown | Self-efficacy; Generalized Self-Efficacy Scale(GSE) |  |
|  |  | Ohira I et al., 2019[24], Japan | 89/238 (10-11 years old; 44.5% girls)  quasi-RCT; primary schools | SEL VS. control; 7 50-min sessions; weekly | Anxiety; The Spence Children’s Anxiety Scale (SCAS) |  |
|  |  | Schwager S et al., 2019 [25], German | 524/939 (10-17 years old; 56% girls)  cluster-RCT; secondary schools | SEL VS. control; 10 weeks; 15 exercises within 10 weeks | Self-efficacy; German Generalized Self-Efficacy Scale  Mental and physical wellbeing; The mental and physical wellbeing subscales of the in Germany well-established KINDL-R Questionnaire |  |
|  |  | Filella G et al., 2018 [26], Spain | 472/903 (Mean age = 12.63 years old; 47.8% girls)  quasi-RCT; secondary schools | SEL VS. control; not shown; weekly | Anxiety; Stait-Trait Anxiety Inventory (STAI) | The intervention showed negative effects on student mental health outcomes. |
|  |  | Allara E et al., 2019 [27], Italy | 1766/3476 (12-13 years old; 50% girls)  quasi-RCT; middle schools | SEL VS. control; 5 lessons in length of 2-4 hours; not shown | Subjective well-being; WHO/Europe Health Behavior in School-aged Children (HBSC) Symptom Checklist |  |
|  | Life skills training curriculum (LST) | McMullen JD et al., 2018 [28], UK | 92/170 (13-18 years old; 49.4% girls)  cluster-RCT; secondary schools | LST VS. control; 1 school year (45-60 min); 24 lessons in a school year | Self-efficacy; The General Self-Efficacy Scale (GES）  Emotional and behavioral problems; The African Youth Psychosocial Assessment Instrument (AYPA） | The interventions showed positive effects on student mental health outcomes. |
|  |  | Fernández-Martínez I et al., 2019 [29], Spain | 67/123 (6 to 8 years old; 44.7% girls)  cluster-RCT; primary schools | LST VS. control; 8 weeks (45min); weekly | Anxiety and depression; Spence Children’s Anxiety Scale-Parent version (SCAS-P) |  |
|  |  | Moulier V et al., 2019 [30], France | 317/413 (10-15 years old; 45.3% girls)  quasi-RCT; middle schools | LST VS. control; 2 weeks (3 hours); 3 sessions over 2 weeks | Self-esteem：the Self-Esteem Scale of Toulouse (ETES)  Well-being; An unvalidated Visual Analog Scale (VAS) |  |
|  |  | Jafarigiv S et al., 2019 [31], Iran | 47/96 (7^th^ grade; 100% girls)  cluster-RCT; secondary schools | LST VS. control; 5 sessions; not shown | Self-esteem; Coopersmith’s Self-esteem Questionnaire  Self-efficacy; Sherer's Self-efficacy Questionnaire |  |
|  |  | Lee M-J et al., 2020 [32], China | 1234/2552 (10-12 years old; 46.7% girls)  cluster-RCT; primary schools | LST VS. usual curricula; 18 months (45 min); not shown | Depressive Affect, positive Affect; Center for epidemiologic studies depression scale for children (CESDC) |  |
|  | Stress management skill training | Heizomi H et al., 2020 [33], Iran | 145/284 (Mean age = 14 years old; 100% girls)  RCT; secondary schools | Stress management skill training VS. control; 2 months (45-60 min); weekly | Psychological well-being; Te Persian versions of General Health Questionnaire (GHQ-28)  Happiness; Oxford Happiness Questionnaire  Self-efficacy; Sherer’s General Self-efficacy Scale  Perceived stress; Cohen’s Perceived Stress Scale | The interventions showed positive effects on student mental health outcomes. |
|  |  | Berger R et al., 2018 [34], Israel | 95/183 (11-14 years old; 50.8% girls)  cluster-RCT; primary schools | Stress reduction curriculum VS. social study curriculum; 16 sessions (45min); two sessions per week | Anxiety was assessed using the Spence Anxiety scale for children (SCAS) |  |
|  |  | Khanna P et al., 2021 [35], India | 60 (stress management training) & 60 (gratitude journaling) & 58 (SMT+GJ)/238 (11-14 years old; 36% girls)  cluster-RCT; middle schools | Stress management training VS. Gratitude journaling VS. SMT+GJ VS. control; 8 weeks; weekly | Well-Being; Mental Health Continuum – Short Form (MHC – SF) |  |
|  | Mindfulness-based curriculum | Fung J et al., 2019[36], USA | 79/145 (13-15 years old; 67.6% girls)  RCT; high school students with elevated mood symptoms | Mindfulness VS. control; 48 50-min sessions during 2 years; weekly | Behavior problems; Youth Self Report (YSR)  Perceived Stress; Perceived Stress Scale (PSS) | The interventions showed positive effects on student mental health outcomes. |
|  |  | Devcich DA et al., 2017 [37], New Zealand | 45/91 (9-11 years old; 42.9% girls)  cluster-RCT; primary schools | Mindfulness VS. Emotional literacy program; 8 weeks (60 min); weekly | Well-being; The Stirling Children’s Wellbeing Scale (SCWBS) |  |
|  |  | Sanger KL et al., 2017 [38], UK | 19/40 (Mean age = 16.8 years old; not shown)  quasi-RCT; not shown | Mindfulness-based curriculum VS. control; 8 weeks (50 minutes); weekly | Well-being; The World Health Organization, Well-Being Index 5-item version (WHO-5) |  |
|  |  | de Carvalho JS et al., 2017 [39], Portugal | 223/454 (Mean age =8.5 years old; 51.5% girls)  quasi-RCT; primary schools | Mindfulness curriculum VS. control; two different stages of the school year (45-60 min)； weekly | Positive and negative affect; a Portuguese version of the PANAS-C |  |
|  |  | Kang Y et al., 2018 [40], USA | 52/100 (mean age = 11.79 years old; 46% girls)  cluster-RCT; primary schools | Mindfulness integrated into Asian history class VS. African history class without mindfulness; 6 weeks (5 min/class); 4-5 times a week | Affect/emotional wellbeing; A modified version of the 20-item Spielberger Anxiety Inventory-Child version (STAI-C) |  |
|  |  | Wright KM et al., 2019 [41], Australia | 44/89 (8–13 years old; 50.5% girls)  RCT; primary school students with internalizing symptoms | Mindfulness-Based Cognitive Therapy VS. cognitive behavior therapy; 10 weeks (90 min); weekly | Mental Health Difficulties; The Revised Child Anxiety and Depression Scale (RCADS), the Self-Description Questionnaire (SDQ) |  |
|  |  | Ghiroldi S et al., 2020 [42], Italy | 232/400 (mean age=8.5 years old; 46.5% girls)  cluster-RCT; primary schools | Mindfulness-based curriculum VS. usual classes; 3-4 months (1 hour); 12 sessions in 3-4 months | Emotional and behavioral problems：Teacher’s Report Form (TRF/6-18) from the Achenbach System of Empirically Based Assessment ((ASEBA ) |  |
|  |  | Lam K et al., 2020 [43], China | 53/115 (11-15 years old; 37% girls)  cluster-RCT; secondary schools | Mindfulness-based curriculum VS. usual lessons; 5 months (70 minutes); monthly | Internalizing and Attention Problems; The Youth Self-Report(YSR) |  |
|  |  | Lee RLT et al., 2020 [44], China | 18/38 (4-6 years old; 44.7% girls)  cluster-RCT; kindergartens | Free play & mindfulness intervention VS. control; 5 days (45 minutes); daily | Emotional Wellbeing; The Smiley Face Likert Scale |  |
|  |  | Volanen SM et al., 2020 [45], Finland | 1646/3519 (12-15 years old; 49.7% girls)  cluster-RCT; middle schools | Mindfulness curriculum VS. active control group VS. inactive control group; 9 weeks (45 min); weekly | Depression; The Beck Depression Inventory (RBDI) |  |
|  |  | Amundsen R et al., 2020 [46], UK | 64/108 (9-10 years old; 49% girls)  quasi-RCT; primary schools | Mindfulness-based curriculum VS. control; 6 weeks (1 h); weekly | Psychological and subjective wellbeing; Sterling Children’s wellbeing scale (SCWBS) |  |
|  |  | Johnson C et al., 2017 [47], Australia | 377/555 (11-16 years old; 45.4% girls)  cluster-RCT; secondary schools | Mindfulness VS. usual curriculum; 9 lessons (40-60 min); weekly | Anxiety and depression; Depression Anxiety Stress Scale – Short form (DASS-21) | The interventions showed no significant effects on student mental health outcomes. |
|  | Resilience-based curriculum | Dray J et al., 2017 [48], Australia | 1255/2105 (12-16 years old; 50.0% girls)  cluster-RCT; secondary schools | resilience-based content in usual curricula VS. usual curricula; 3 years; not shown | Mental health problems; The Strengths and Difficulties Questionnaire (SDQ) | The interventions showed no significant effects on student mental health outcomes. |
|  |  | Kozina A et al., 2020 [49], Slovenia | 44/80 (13-14 years old; 53.8% girls)  cluster-RCT; secondary schools | Resilience-based training curriculum VS. control; 10 weeks (30-60 min); weekly | Anxiety; The AN-UD anxiety scale  Behavioral problems; Strengths and Difficulties Questionnaire | The interventions showed positive effects on student mental health outcomes. |
|  |  | Olowokere AE et al., 2018 [50], Nigeria | 176/339 (Mean age= 13.53 years old; 47.2% girls)  cluster-RCT; vulnerable high school students | resilience-based training curriculum VS. peer support group; 6 weeks (2 h); weekly | Anxiety; Spence’s children anxiety scale Depression; Center for epidemiological studies depression scale for Children Self-esteem; Rosenberg self-esteem scales |  |
|  | Game play | Perry Y et al., 2017 [51], Australia | 242/540 (Mean age = 16.7 years old; 63.1% girls)  cluster-RCT; secondary schools | Game play (SPARX-R) VS. control; 5 weeks (20-30 min); 1 to 2 modules per week | Depression; The Major Depression Inventory  Anxiety; The Spence Children’s Anxiety Scale (SCAS)） | The interventions showed positive effects on student mental health outcomes. |
|  |  | Streimann K et al., 2019 [52]，Estonia | 362/708 (mean age=7.1 years old; 50.1% girls)  cluster-RCT; primary schools | PAX Good Behavior Game VS. control; 1 year; not shown | Mental health problems; The teacher-rated Strengths and Difficulties Questionnaire (SDQ) |  |
|  | Cognitive Behavioral Therapy (CBT) | Martinsen KD et al., 2021[53], Norway | 358/795 (8-12 years old; 58.0% girls)  cluster-RCT; children with elevated levels of anxious and/or depressive symptoms | CBT-based curriculum VS. control; 10 weeks; twice a week | Self-esteem; The Beck Self-Concept Inventory for Youth (BSCI-Y II) | The interventions showed positive effects on student mental health outcomes. |
|  |  | Garmy P et al., 2019 [54], Sweden | 462/948 (13-15 years old; 62.3% girls)  cluster-RCT; secondary schools | CBT-based curriculum VS. control; 10 weeks (1.5 hour); weekly | Depression; The Center for Epidemiological Studies Depression Scale (CES-D) |  |
|  |  | Redfern A et al., 2019 [55], UK | 638/698 (7-10 years old; not shown)  quasi-RCT; primary school children with mental health vulnerability | CBT-based curriculum VS. control; 8 weeks (1 hour); weekly | Well-being/distress; The Children’s Outcome Rating Scale (CORS) Emotional /behavioral difficulties; Me and My Feelings |  |
|  |  | Terry JD et al., 2020 [56], USA | 22/43 (not shown; 39.53% girls)  RCT; middle school students displaying poor academic performance and/or disruptive behavior | Motivational Interviewing & CBT-based curriculum VS. control; not shown; not shown | Self-Efficacy; The Children’s Perceived Self-Efficacy (CPSE) |  |
|  |  | de la Torre-Luque A et al., 2020 [57], Spain | 21/61 (Mean age = 13.76 years old; 54.7% girls)  RCT; adolescents reporting key symptoms of anxiety disorders | CBT-based curriculum VS. school-work programme VS. control; 8 weeks (1 hour); weekly | Anxiety; Mini-International Neuropsychiatric Interview for Children and Adolescents, Revised Child Anxiety and Depression Scale (RCADS) |  |
|  |  | Lowe C et al., 2021 [58], Australia | 28/56 (17-18 years old; 77% girls)  RCT; the final year of high school | CBT-based curriculum VS. usual care; 8 weeks; not shown | Depression, anxiety and stress; The Depression Anxiety and Stress Scale 21  Positive and negative characteristics; Strengths and Difficulties Questionnaire (SDQ)-Teacher Version |  |
|  |  | Klim-Conforti P et al., 2021 [59], Canada | 200/430 (11-14 years old; 61.6% girls)  cluster-RCT; junior high schools | Harry Potter-based CBT curriculum curriculum VS. control; 3 months; not shown | Suicidality scores; The Life Problems Inventory (LPI)  Anxiety and depression; the Revised Child Anxiety and Depression Scale (RCADS) |  |
|  |  | Ahlen J et al., 2019 [60], Sweden | 353/695 (8-11 years old; 48% girls)  cluster-RCT; primary schools | CBT-based curriculum VS. usual classes; 10 weeks (60 min); weekly | Anxiety; The Spence Children’s Anxiety Scale (SCAS) Depression; The Children’s Depression Inventory-Short Version (CDI-S) | The interventions showed no significant effects on student mental health outcomes. |
|  | CBT & Game play | Poppelaars M et al., 2016 [61], Netherlands | 50 (Op Volle Kracht [OVK]) & 51 (SPARX) & 56 (OVK+SPARX)/208 (11-16 years old; 100% girls)  RCT; secondary school girls with elevated depressive symptoms | CBT (OVK) VS. game play (SPARX) VS. OVK & SPARX VS. control; 5 months; OVK:1 hour per week/ SPARX:20-40 minutes per week | Depression; Reynolds Adolescent Depression Scale (RADS-2) | The interventions showed positive effects on student mental health outcomes. |
|  | CBT & Attention regulation training | Waters AM et al., 2019 [62], Australia | 116 (Positive Search Training) & 127 (Cognitive Behavioral Intervention) /303 (7-11 years old; 48.5% girls)  cluster-RCT; primary schools | Attention regulation training (PST) VS. (CBI) VS. usual curriculum; eight 30-min sessions during 4 weeks; twice a week | Anxiety; The Spence Children’s Anxiety Scale (SCAS) | The interventions showed positive effects on student mental health outcomes. |
|  | Outdoor learning curriculum | Bolling M et al., 2019 [63], Denmark | 511/631 (9-13 years old; 53.4% girls)  quasi-RCT; primary schools | Education outside the classroom (EOtC) for 2.4-6.7 hours per week VS. EotC for 0.0-1.9 hours per week; not shown; not shown | Psychosocial well-being (emotional problems, conduct problems, et al.); Strengths and Difficulties Questionnaire (SDQ) | The interventions showed positive effects on student mental health outcomes. |
|  |  | Harvey DJ et al., 2020 [64], UK | 329/404 (8-11 years old; 57.2% girls)  quasi-RCT; primary schools | Outdoor education VS. control; 1 academic year; seven sessions per term | Well being; The KIDSCREEN-27, the  adapted version of the Positive and Negative Affect Schedule for children (PANAS-C) scale |  |
| Interpersonal relationship | Teacher classroom management training | Ford T et al., 2017 [65], UK | 1037/2075 (1-4^th^ grade; 46.9% girls)  cluster-RCT; primary schools | Teacher classroom management training VS. Teaching as usual; not shown; not shown | The Strengths and Difficulties Questionnaire (SDQ) | The interventions showed positive effects on student mental health outcomes. |
|  |  | Neal SC et al., 2020 [66], USA | 485/939 (4 years old; 48.3% girls)  cluster-RCT; kindergartens | Teacher classroom management training VS. control; not shown; not shown | Internalizing and externalizing behaviors |  |
|  | Interpersonal psychotherapy–adolescent skills training | Benas JS et al., 2019 [67], USA | 95/186 (Mean age = 14.01 years old; 66.7% girls)  RCT; secondary schools | Interpersonal psychotherapy–adolescent skills training VS. group counseling; at least 10 sessions; not shown | Internalizing and externalizing problems; ASEBA scales, Youth Self-Report [YSR], Child Behavior Checklist [CBCL], Teacher’s Report Form [TRF] |  |
|  | Small-group learning | Van Ryzin MJ et al., 2020 [68], USA | 875/1890 (not shown; 47.1% girls)  cluster-RCT; middle schools | Structured small-group learning activities VS. control; 2 school years; not shown | Emotional problems; The Emotional Problems subscale of the Strengths and Difficulties Questionnaire |  |
| Homework and tests | CBT | Putwain DW et al., 2018 [69], UK | 25/56 (Mean age=14.7 years old; 66.1% girls)  RCT; secondary school students preparing for high-stakes tests | CBT VS. control; 6 weeks (40 minutes); weekly | Test anxiety; The 20-item Revised Test Anxiety Scale | The interventions showed positive effects on student mental health outcomes. |
|  | Attention training | Fergus TA et al., 2019 [70], USA | 39/73 (8 th grade; 65.8 girls)  cluster-RCT; middle schools | Attention training VS. music listening; 1 week; daily | Test Anxiety; Children’s Test Anxiety Scale (CTAS) |  |
| Physical activity in school | Physical activity in the classroom setting | Rizal H et al., 2019 [71], Malaysia | 177/322 (10-11 years old; 50.6% girls)  quasi-RCT; primary schools | Brain breaks physical activity in the classroom VS. control; 12 weeks (30 min); weekly | Self-efficacy; The three-factor, 18-item self-efficacy scale originated from Bandura | The interventions showed positive effects on student mental health outcomes. |
|  |  | Cornelius C et al., 2020 [72], USA | 50/114 (Mean age = 16 years old; 33% girls)  quasi-RCT; secondary schools | pedal desks during class time vs. control; pedal at least 10 min for each class period during 14 weeks; 3 days a week | Self-efficacy; The Self-Efficacy Scale and the Self-Efficacy for Exercise scale |  |
|  |  | Mok MMC et al., 2020 [73], Croatia, Lithuania, Macedonia, Poland, Romania, Serbia, South Africa, and Turkey | 1914/3036 (8-11 years old; 50.7% girls)  quasi-RCT; primary schools | group activity exercise within the classroom VS. control; 4 months; 3– 5 minutes in length, twice per day, 5 days each week | Self-efficacy; The Attitudes toward Physical Activity Scale (APAS) |  |
|  |  | Sherry AP et al., 2020 [74], UK | 22/49 (9-10 years old; 52.1% girls)  quasi-RCT; primary schools | Adjustable sit–stand desks VS. traditional furniture; 8 months (first 20 min of a mathematics class); not shown | Behavior-related mental health; The 25-item Strength and Difficulties questionnaire |  |
|  | Physical education (PE) lessons | Costigan SA et al., 2016 [75], Australia | 21 (aerobic exercise) & 22 (resistance and aerobic exercise)/65 (14-16 years old; 30.8% girls)  RCT; secondary schools | aerobic exercise VS. resistance and aerobic exercise VS. control; 8 weeks (8-10 min); 3 times per week | Psychological well-being; The Flourishing Scale  Psychological distress; The Kessler Psychological Distress Scale | The interventions showed positive effects on student mental health outcomes. |
|  |  | Ruiz-Ariza A et al., 2019 [76], Spain | 94/184 (12–16 years old; 46.7% girls)  RCT; secondary schools | Cooperative high-intensity interval training VS. static stretching; 12 weeks (16 min); two sessions per week | Well-being; The Trait and Emotional Intelligence Questionnaire Short Form (TEIQue-SF) |  |
|  |  | Cocca A et al., 2020 [77], Australia | 102/229 (mean age=10.24; 47.2% girls)  quasi-RCT; primary schools | Game-based PE lessons VS. traditional PE activities; 6 months (45min); twice a week | Psychological health; Psychological Wellbeing Questionnaire (PWBQ), Rosenberg self-esteem scale (RSES) |  |
|  |  | Andrade A et al., 2020 [78], Brazil | 68/140 (7-11 years old; 57.9% girls)  cluster-RCT; primary schools | Exergames in PE lessons VS. traditional PE lessons; 2 weeks (40minutes); 3 sessions during 2 weeks | Mood; Brunel mood scale  Self-esteem; Rosenberg self-esteem scale |  |
|  |  | Zhang Y et al., 2020 [79],China | 24/51 (12 to 13 years old; 52.9% girls)  RCT; junior middle schools | 5 indoor courses & 3 outdoor basketball matches VS. usual curricula; 8 weeks (45 min); weekly | Self-efficacy; 18 items to evaluate participants’ confidence in doing physical activity and changing PA-related behavior |  |
|  |  | Koszałka-Silska A et al., 2021 [80], Poland | 30/70 (15-16 years old; 0% girls)  quasi-RCT; high schools | Adventure education (AE) programme in PE class VS. usual PE class; 12 weeks (2*45min); weekly | Self-esteem; The Polish version of the Rosenberg’s Self-Esteem Scale |  |
|  |  | Resaland GK et al., 2018 [81], Norway | 596/1129 (10 years old; 47.9% girls)  cluster-RCT; primary schools | 300 min of physical activity per week VS. 135 min per week; 7 months; 300 min per week | Psychological well-being; the Kidscreen-27 questionnaire | The interventions showed no significant effects on student mental health outcomes. |
|  |  | Smith JJ et al., 2018 [82], Australia | 296/508 (Mean age = 14.1 years old; 49.6% girls)  cluster-RCT; Secondary schools | Resistance training VS. control; 10 weeks; 100 min per week | Global self-esteem; A5-item subscale from the short-form of the Physical Self-Description Questionnaire (PSDQ) Subjective well-being; Diener and colleagues’ psychological flourishing scale |  |
|  |  | Luna P et al., 2019 [83], Spain | 69/113 (12-15 years old; 43% girls)  cluster-RCT; secondary schools | Physical-sport programme VS. traditional collective sport with a conventional teaching style; 6 weeks (55 min); 2–3 sessions per week | Anxiety; The Social Anxiety Scale for Adolescents (SAS-A) |  |
|  |  | Gall S et al., 2020 [84], Switzerland | 310/758 (8-13 years old; 49.2% girls)  cluster-RCT; disadvantaged primary schools | physical activity intervention (PE lessons; aerobic dancing-to- music lessons; regular in-class physical activity breaks) VS. control; 20 weeks; 2 PE lessons and 1 dancing-to- music lessons per week | Psychological well-being; The subscale of the 27-item KIDSCREEN questionnaires |  |
|  | Yoga | Halliwell E et al., 2018 [85], UK | 190/344 (9-11 years old; 54.4% girls)  cluster-RCT; primary schools | Yoga VS. regularly scheduled physical education lesson; 40 minutes; weekly | Positive and negative affect; The Positive and Negative Affect Scale for Children | The interventions showed positive effects on student mental health outcomes. |
|  |  | Shreve M et al., 2021 [86], USA | 69/71(8-10 years old; 8-10 years old)  quasi-RCT; primary schools | Yoga VS. control; 8 weeks (10 min); not shown | Anxiety; the Screen for Child Anxiety Related Emotional Disorders (SCARED) |  |
|  | Active learning & PE | Avitsland A et al., 2020 [87], Norway | 847 (“Active learning”) & 717 (“Don’t worry, be happy”)/2073 (14-15 years old; 49% girls)  cluster-RCT; secondary schools | “Active learning” VS. “Don’t worry, be happy” VS. control; 29 weeks; weekly | mental health; the Strengths and Difficulties Questionnaire (SDQ) | The interventions showed positive effects on student mental health outcomes. |
|  |  | Schmidt SK et al., 2020 [88], Norway | 197/644 (13-15 years old; 49.8% girls)  quasi-RCT; secondary schools | 120 min/week of physically active learning (PAL) and 25 min/week of physical active breaks VS. control; 7 months; weekly | Psychological well-being; The subscale of the 27-item KIDSCREEN questionnaires |  |
| After-school activities | Sports | Annesi JJ et al., 2016 [89], USA | 88/145 (9-12 years old; 44.8% girls)  cluster-RCT; primary schools | Moderate-to-vigorous physical activity during outside school hours VS. typical after-school care; 12 weeks (45 min); 4 days per week | Negative mood; The abbreviated version of the Total Mood Disturbance scale was used | The interventions showed positive effects on student mental health outcomes. |
|  |  | Ho FKW et al., 2017 [90], China | 333/664 (mean age =12.3 years old; 58.1% girls)  RCT; high schools | After-school sports mentorship programme VS. control; 18 weekly sports sessions (90 min); weekly | Mental well-being; SF-12v2 |  |
|  |  | Anusuya US et al., 2021[91], India | 30/60 (14-16 years old; 50.0% girls)  RCT; secondary schools | Yoga-based relaxation activity VS. control; 2 weeks (30 minutes); 6 days a week | Anxiety; The Spielberg’s State-Trait Anxiety inventory |  |
|  |  | McMahon K et al., 2021[92], USA | 66/118 (11-14 years old; 52.5% girls)  quasi-RCT; secondary schools | Yoga for youth programme VS. alternate activity; 6 weeks (40min in length); twice a week | Depression and anxiety; Depression Anxiety Stress Scale-21 (DASS-21) | The interventions showed no significant effects on student mental health outcomes. |
|  | Clubs | Jayman M et al., 2019 [93], UK | 66/126 (11-14 years old; 58.7% girls)  quasi-RCT; secondary school students with emotional symptoms | Pyramid Club VS. control; 10 weeks (90 min); weekly | Socio-emotional well-being; The Strengths and Difficulties Questionnaire (SDQ) | The interventions showed positive effects on student mental health outcomes. |
|  | Arts lessons | Duberg A et al., 2020 [94], Sweden | 59/112 (13 to 18 years old; 100% girls)  RCT; high school girls with with stress-related somatic symptoms and emotional distress | Dance curriculum VS. control; 8 months (75 min); twice a week | Emotional distress; The Swedish survey ‘Life and Health – Young People', and are in line with the type of questions used in the ‘Health Behavior in School-aged Children’ (HBSC) |  |
|  |  | Kim H-S et al., 2018[95], South Korea | 30/60 (7-12 years old; 56.7% girls)  RCT; primary schools | Flute performance lessons VS. control; 24 weeks (50 min); weekly | Anxiety; The 20-item trait anxiety scale from the State–Trait Anxiety Inventory | The interventions showed no significant effects on student mental health outcomes. |
| Multi-component  (Curriculum & Interpersonal relationship, etc.) | A whole-school, multi-component intervention | Shinde S et al., 2020 [96], India | 2854 (SEHER Mitra [SM]) & 2285 (Teacher as SEHER Mitra [TSM]) /7824 (13-15 years old; 45% girls)  cluster-RCT; secondary schools | Social-emotional learning curriculum & peer group activity, etc. VS. control; 2 years; not shown | Depression; Patient Health Questionnaire-9 (PHQ-9) | The interventions showed positive effects on student mental health outcomes. |
|  |  | Shinde S et al., 2018 [97], India | 5316 (SM) & 4475 (TSM)/14414 (13-14 years old; not shown)  cluster-RCT; secondary schools | Social-emotional learning curriculum & peer group activity, etc. VS. control; 1 year; not shown | Depression; Patient Health Questionnaire-9 (PHQ-9) |  |
|  |  | Singla DR et al., 2020 [98], India | 2854/5539 (13-14 years old; 51.5% girls)  cluster-RCT; secondary schools | Social-emotional learning curriculum & peer group activity, etc. VS. control; two full academic calendar years; not shown | Depression; Patient Health Questionnaire-9 (PHQ-9) |  |
|  |  | Kiviruusu O et al., 2016 [99], Finland | 2036/3704 (7-9 years old; 51.4% girls)  cluster-RCT; primary schools | Social-emotional learning curriculum & small-group learning, etc. VS. control; two school terms; not shown | Psychological problems; The Strengths and Difficulties Questionnaire (SDQ) |  |

**Table S3: Summaries of observational research on relationships between school-related factors and student mental health outcomes (n=98).**

| **Category** | **Study design** | **Author, year, country** | **Description of school-related factors** | **N** | **Age range** | **Psychological ill-being ^a^** | **Psychological well-being ^a^** |
| --- | --- | --- | --- | --- | --- | --- | --- |
| Curriculum | Corss-sectional | Jansen M et al., 2019 [100], German | Science course composition | 4698 | 15.02±0.66 |  | + |
|  |  | Nagamitsu S et al., 2020 [101], Japan | Academic course | 22419 | 13-18 | + |  |
|  |  | Rodriguez S et al., 2020 [102], Spain | Success in mathematics | 897 | 9-13 | - |  |
|  |  | Holopainen L et al., 2020 [103], Finland | Difficulties in academic lessons | 464 | 16 |  | - |
| Homework | Cross-sectional | Yockey RA et al., 2019 [104], USA | Not doing required homework | 2138 | 12-17 | + |  |
|  |  |  | Extracurricular activities |  |  | - |  |
|  |  | Lonnfjord V et al., 2020 [105], Sweden | Demanding homework | 2004 | 13-15 | + |  |
|  |  | Yeo SC et al., 2020 [106], Singapore | Homework duration | 1225 | 13-18 | + |  |
|  |  | Lv B et al., 2021 [107], China | Homework duration | 9312 | 15.07±1.04 | + |  |
|  | Longitudinal | Hamer M et al., 2016 [108], UK | Homework duration | 2038 | 16 | + |  |
| Tests | Cross-sectional | Al-Qahtani AM et al., 2017[109], Saudi Arabia | School's examinations | 376 | 1-3^rd^ grade in secondary schools | + |  |
|  |  | Malak MZ et al., 2017 [110], Jordan | School's examinations (school class) | 800 | 12-18 | + |  |
|  |  | Sarı SA et al., 2017 [111], Turkey | Test anxiety | 724 | 16-18 |  | - |
|  |  | Cikrikci O et al., 2019 [112], Turkey | Test anxiety | 397 | 14-18 |  | - |
|  |  | Long E et al., 2020 [113], 2020, UK | Exam pressure | 2571 | 15-16 | + |  |
|  |  | Haerens L et al., 2019 [114], Belgium | Knowledge about the criteria for an upcoming test | 659 | 14.72 | ○ |  |
|  |  | Nair S et al., 2017 [115], India | Failure in examinations | 693 | 13-17 | + |  |
|  |  |  | Punishment in form of more homework |  |  | + |  |
|  |  | Bashir MBA et al., 2019 [116], Sudan | Test scores | 388 | 15-18 | - |  |
|  |  | Evensen M et al., 2019 [117], Norway | Test scores | 4202 | 6-16 | - |  |
|  |  | Torrano R et al., 2020 [118], Spain | Test scores | 1181 | 12-18 | - |  |
| Interpersonal relationships | Cross-sectional | Warne M et al., 2017 [119], Sweden | Positive interpersonal relationships | 1527 | 12-16 |  | + |
|  |  | Oberle E et al., 2018 [120], Canada | Positive interpersonal relationships | 406 | 11.27 |  | + |
|  |  | Povedano-Diaz A et al., 2019 [121], Spain | Positive interpersonal relationships | 2373 | 11-18 |  | + |
|  |  | Yun JY et al., 2019 [122], Korea | Positive interpersonal relationships | 1991 | 15.3±1.7 | - |  |
|  |  | Tong L et al., 2019 [123], China | Positive interpersonal relationship | 1369 | 10^th^ grade | - |  |
|  |  | Herres J et al., 2016 [124], USA | Negative interpersonal events | 132 | 14 | + |  |
|  |  | Adewuya AO et al., 2020 [125], Nigeria | Negative interpersonal relationships | 9441 | 15.61±1.49 | + |  |
|  |  | He GH et al., 2019[126], China | Negative interpersonal relationships | 6576 | 13.37±1.84 | + |  |
|  |  | Li J et al., 2020 [127], China | Negative interpersonal relationships | 10131 | 13-18 | + |  |
|  |  | Fernandez-Sogorb A et al., 2021 [128], Spain | Negative interpersonal relationships | 756 | 9.6±1.12 | + |  |
|  | Longitudinal | Burns EC et al., 2018 [129], Australia | Positive interpersonal relationships | 1481 | 8-10^th^ grade |  | + |
|  |  | Weyns T et al., 2019 [130], Belgium | Negative teaching behavior | 237 | 5.19 | + |  |
|  |  | Pössel P et al., 2018[131], USA | Teacher support | 1452 | 11-16 | - |  |
| Interpersonal relationships: Teacher-student | Cross-sectional | Barnard AD et al., 2017 [132], USA | Negative teaching behavior | 777 | 3-4^th^ grade | + | ○ |
|  |  |  | Social-emotional teaching behavior |  |  |  | + |
|  |  | Cauley B et al., 2017 [133], USA | Negative teaching behavior | 968 | 9-12^th^ grade |  | - |
|  |  |  | Social-emotional teaching behavior |  |  |  | + |
|  |  | Mizuta A et al., 2017 [134], Japan | Teacher support | 2466 | 12-15 | - |  |
|  |  | Quin D et al., 2017 [135], Australia | Teacher support | 88 | 12.8 | - |  |
|  |  | Guo C et al., 2018 [136], China | Teacher support | 5399 | 8-10^th^ grade | + |  |
|  |  | Bennefield Z, 2018 [137], USA | Teacher support | 10148 | 13-18 |  | + |
|  |  | Corder K et al., 2020 [138], UK | Teacher support | 671 | 13-14 |  | + |
|  |  | Lan X et al., 2019 [139], China | Teacher support | 843 | 13-18 | - | + |
|  |  | Phan HP et al., 2020 [140], China | Positive relationship | 760 | 16-18 |  | + |
|  |  | Pereyra SB et al., 2020 [141], USA | Positive relationship | 2214 | 14-18 | - |  |
|  | Longitudinal | Kurdi V et al., 2017 [142], Canada | Negative events with teachers | 350 | 3-4^th^ grade | + (girls more susceptible) |  |
|  |  | Shukla KD et al., 2020 [143], India | Negative teaching behavior | 6423 | 6-8^th^ grade |  | - |
|  |  | Wang C et al., 2016 [144], Australia | Teacher-student closeness | 2857 | 6-7 | - |  |
|  |  | Nie Q et al., 2019 [145], China | Teacher-student closeness | 1108 | 14-18 |  | + |
|  |  | Wong MD et al., 2021 [146], USA | Respect for Teacher | 1114 | 9-11^th^ grade | - | + |
| Interpersonal relationships: student-student | Cross-sectional | Xavier A et al., 2018 [147], Portugal | Peer hassles | 776 | 12-18 | + |  |
|  |  | Lessard LM et al., 2018 [148],USA | Friendlessness | 5991 | 6-8^th^ grade | + |  |
|  |  | Pandey AR et al., 2019 [149], Nepal | Friendlessness | 6531 | 11-18 | + |  |
|  |  | Troop-Gordon W et al., 2019 [150], USA | Negative peer beliefs | 366 | 9.34 ± 0.07 | + |  |
|  |  | Humenny G et al., 2021[151], Poland | Negative network | 388 | 12-13 | + |  |
|  |  | Biswas T et al., 2020 [152], Australia | Peer conflict and isolation | 275057 | 12-17 | + |  |
|  |  | Schmidt A et al., 2020 [153], German | Relationship frustration | 119,90,108 | 9-12 | + |  |
|  |  |  | Relationship satisfaction |  |  |  | + |
|  |  | Carter R et al., 2017 [154], USA | Same-race peers and friends | 607 | 13-17 | ○ |  |
|  |  | Attar-Schwartz S et al., 2019[155], Canada | Classmates’ support | 243 | 7^th^ grade |  | - |
|  |  | Baytemir K et al., 2019 [156], Turkey | Friendship closeness | 268 | 14-18 |  | + |
|  |  | Wu N et al., 2019 [157], China | Peer acceptance | 813 | 11-16 | - |  |
|  |  | Copeland M et al., 2019 [158], USA | Peer networks (more friends) | 11160 | 11-12^th^ grade | - |  |
|  |  | Dang J et al., 2019 [159], China | Class entitativity | 408 | 12-17 |  | + |
|  |  | Lyell KM et al., 2020 [160], USA | Peer support | 364 | ＞12 | - | + |
|  | Longitudinal | Fussner LM et al., 2018 [161], USA | Peer rejection | 133 | 8-13 | + |  |
|  |  | Zhang S et al., 2018 [162], Netherlands | Conflicts with friends | 1126 | 11-18 | + |  |
|  |  | Mali LV et al., 2019 [163], USA | Unpopularity among students | 393 | 14-17 | + |  |
|  |  | Martinez G et al., 2019 [164], Mexico | Peer stressors | 338 | 7-10^th^ grade | + |  |
|  |  | Delgado MY et al., 2019 [165], USA | Peer discrimination | 246 | 12.55 | + |  |
|  |  |  | Friendship Intimacy |  |  | - |  |
|  |  | Laetsch A et al., 2017 [166], German | helpfulness in class | 1088 | 13.70 | - | + |
|  |  |  | competition in class |  |  | + |  |
|  |  | Wood MA et al., 2017 [167], Canada | Friendship durability | 380 | 10-13 | - |  |
|  |  | van Harmelen AL et al., [168]2016,UK | Friendship support | 771 | 14 | - |  |
|  |  | King RB et al., 2017 [169], China | Class-level happiness | 676 | 14.84 |  | + |
|  |  | Ng-Knight T et al., 2019 [170], UK | Friendship stability | 593 | 11 | - |  |
|  |  | Oberle E et al., 2018 [171], Canada | Peer belonging | 1943 | 8-11 |  | + |
|  |  | Tetzner J et al., 2019 [172], German | Peer acceptance | 7272 | 14.1 |  | + |
|  |  | Chen Y et al., 2019 [173], China | Peer acceptance | 1295 | 12.86 |  | + |
|  |  | Yang Y et al., 2020 [174], China | Peer acceptance, support | 1928 | 6-9^th^ grade | - |  |
| Physical activity in school | Cross-sectional | Farren GL et al., 2018 [175], USA | Physical activity | 249 | 12.85 ± 0.89 | - |  |
|  |  | Belton S et al., 2019 [176], Ireland | Physical activity | 535 | 12-14 |  | + |
|  |  | Barth Vedøy I et al., 2020[177], Norway | Physical activity | 599 | 13-16 |  | + |
|  |  | Wunsch K et al., 2021 [178], German | Physical activity | 1565 | 11-17 |  | + |
|  |  | Park S et al., 2020 [179], South Korea | Participation in Physical Education | 28451 | 16.4 | - |  |
|  | Longitudinal | Bell SL et al., 2019 [180], UK | Physical activity | 794 | 12-13 | - |  |
|  |  | Opdal IM et al., 2019 [181], Norway | Physical activity | 676 | 15-17 | ○ |  |
| After-school activities | Cross-sectional | Hyakutake A et al., 2016 [182], Japan | Leisure-time physical activity | 241 | 13.6 ± 0.9 | - |  |
|  |  | McMahon EM et al., 2017 [183], ten European countries | Leisure-time physical activity | 11072 | 14.8±0.84 | - | + |
|  |  | Tajik E et al., 2017 [184], Malaysia | Leisure-time physical activity | 1747 | 13-14 | - |  |
|  |  | Kleppang AL et al., 2019 [185], Norway | Leisure-time physical activity | 1330 | 15-16 | - |  |
|  |  | Bélair MA et al., 2018 [186], Canada | Leisure-time physical activity | 9702 | 14-15 | - |  |
|  |  | O'Brien K et al., 2020 [187], Australia | Leisure-time physical activity | 14136 | 5.27 | - |  |
|  |  | Guddal MH et al., 2019 [188], Norway | Leisure-time physical activity | 7619 | 13-18 | - | + |
|  |  | Oosterhoff B et al., 2017 [189], USA | Sports | 10148 | 13-18 |  | + |
|  |  |  | Clubs/organizations |  |  |  | + |
|  |  |  | Music |  |  |  | + |
|  |  | Reverdito RS et al., 2017 [190], Brazil | Sports participation | 821 | 13.6 ±1.5 |  | + |
|  |  | Kleppang AL et al., 2018 [191], Norway | Sports participation | 5331 | 15-16 | - |  |
|  |  | Oberle E et al., 2020 [192], Canada | Organized activities | 28712 | 12.25±0.51 |  | + |
|  |  | Jiang R et al., 2021 [193], China | Sports participation | 306 | 7-13 |  | + |
|  | Longitudinal | Guzmán-Rocha MD et al., 2017 [194], USA | Organized activities | 660 | 10-12^th^ grade |  | + |
|  |  | Moeijes J et al., 2018 [195], Netherlands | Sports participation | 487 | 11.9±0.5 | - | + |
|  |  | Oberle E et al., 2019 [196], Canada | Organized activities | 10149 | 9.21±0.48 |  | + |
|  |  | Tu HM et al., 2020 [197], China | Sports participation | 483 | 13-18 |  | + |
|  |  |  | Peer support |  |  |  | + |

^a^: classified as positive (+), negative (−) associations and no significant associations (○), depending on the directions of the associations.

**Reference:**

1. Burckhardt R, Manicavasagar V, Batterham PJ, Hadzi-Pavlovic D. A randomized controlled trial of strong minds: A school-based mental health program combining acceptance and commitment therapy and positive psychology. J Sch Psychol. 2016;57:41-52.

2. Coelho VA, Sousa V, Figueira AP. The Effectiveness of a Portuguese Elementary School Social and Emotional Learning Program. J Prim Prev. 2016;37(5):433-47.

3. Coelho VA, Sousa V. Comparing Two Low Middle School Social and Emotional Learning Program Formats: A Multilevel Effectiveness Study. J Youth Adolesc. 2017;46(3):656-67.

4. Coelho VA, Sousa V. Differential Effectiveness of a Middle School Social and Emotional Learning Program: Does Setting Matter? J Youth Adolesc. 2018;47(9):1978-91.

5. Hoang-Minh D, Weiss B, Cao Minh N, Nam T, Pollack A. Vietnam as a case example of school-based mental health services in low and middle income countries: Efficacy and effects of risk status. School Psychology International. 2017;38(1):22-41.

6. Shoshani A, Slone M. Positive Education for Young Children: Effects of a Positive Psychology Intervention for Preschool Children on Subjective Well Being and Learning Behaviors. Frontiers in Psychology. 2017;8.

7. Roberts CM, Kane RT, Rooney RM, Pintabona Y, Baughman N, Hassan S, et al. Efficacy of the Aussie Optimism Program: Promoting Pro-social Behavior and Preventing Suicidality in Primary School Students. A Randomised-Controlled Trial. Frontiers in Psychology. 2018;8.

8. Garaigordobil M, Jaureguizar J, Bernarás E. Evaluation of the effects of a childhood depression prevention program. J Psychol. 2019;153(2):127-40.

9. Flynn D, Joyce M, Weihrauch M, Corcoran P. Innovations in Practice: Dialectical behaviour therapy – skills training for emotional problem solving for adolescents (DBT STEPS-A): evaluation of a pilot implementation in Irish post-primary schools. Child and Adolescent Mental Health. 2018;23(4):376-80.

10. Young JF, Jones JD, Sbrilli MD, Benas JS, Spiro CN, Haimm CA, et al. Long-Term Effects from a School-Based Trial Comparing Interpersonal Psychotherapy-Adolescent Skills Training to Group Counseling. Journal of Clinical Child and Adolescent Psychology. 2019;48:S362-S70.

11. Ab Ghaffar SF, Mohd Sidik S, Ibrahim N, Awang H, Gyanchand Rampal LR. Effect of a School-Based Anxiety Prevention Program among Primary School Children. Int J Environ Res Public Health. 2019;16(24).

12. Dowling K, Simpkin AJ, Barry MM. A Cluster Randomized-Controlled Trial of the MindOut Social and Emotional Learning Program for Disadvantaged Post-Primary School Students. J Youth Adolesc. 2019;48(7):1245-63.

13. Iyer RB, Iyer BN. The Impact of Heartfulness-based Elective on Middle School Students. Am J Health Behav. 2019;43(4):812-23.

14. Zhao Y, Yu F, Wu Y, Zeng G, Peng K. Positive Education Interventions Prevent Depression in Chinese Adolescents. Frontiers in Psychology. 2019;10.

15. Taghvaienia A, Zonobitabar A. Positive intervention for depression and teacher-student relationship in Iranian high school girl students with moderate/mild depression: a pilot randomized controlled trial. Child and Adolescent Psychiatry and Mental Health. 2020;14(1).

16. Johnstone KM, Middleton T, Kemps E, Chen J. A pilot investigation of universal school-based prevention programs for anxiety and depression symptomology in children: A randomized controlled trial. Journal of Clinical Psychology. 2020;76(7):1193-216.

17. Bono G, Mangan S, Fauteux M, Sender J. A new approach to gratitude interventions in high schools that supports student wellbeing. Journal of Positive Psychology. 2020;15(5):657-65.

18. Maalouf FT, Alrojolah L, Ghandour L, Afifi R, Dirani LA, Barrett P, et al. Building Emotional Resilience in Youth in Lebanon: a School-Based Randomized Controlled Trial of the FRIENDS Intervention. Prevention Science. 2020;21(5):650-60.

19. Humphrey N, Panayiotou M. Bounce Back: randomised trial of a brief, school-based group intervention for children with emergent mental health difficulties. European Child & Adolescent Psychiatry. 2020.

20. Sánchez-Sansegundo M, Ferrer-Cascales R, Albaladejo-Blazquez N, Alarcó-Rosales R, Bowes N, Ruiz-Robledillo N. Effectiveness of the reasoning and rehabilitation v2 programme for improving personal and social skills in spanish adolescent students. International Journal of Environmental Research and Public Health. 2020;17(9).

21. Zhu X, Shek DTL. Impact of a positive youth development program on junior high school students in mainland China: A pioneer study. Children and Youth Services Review. 2020;114.

22. Tomyn JD, Fuller-Tyszkiewicz M, Richardson B, Colla L. A Comprehensive Evaluation of a Universal School-Based Depression Prevention Program for Adolescents. J Abnorm Child Psychol. 2016;44(8):1621-33.

23. Schanen JG, Skenandore A, Scow B, Hagen J. Assessing the Impact of a Healthy Relationships Curriculum on Native American Adolescents. Soc Work. 2017;62(3):251-8.

24. Ohira I, Urao Y, Sato Y, Ohtani T, Shimizu E. A pilot and feasibility study of a cognitive behavioural therapy-based anxiety prevention programme for junior high school students in Japan: a quasi-experimental study. Child and Adolescent Psychiatry and Mental Health. 2019;13(1).

25. Schwager S, Berger U, Glaeser A, Strauss B, Wick AK. Evaluation of "Healthy Learning. Together", an Easily Applicable Mental Health Promotion Tool for Students Aged 9 to 18 Years. Int J Environ Res Public Health. 2019;16(3).

26. Filella G, Ros-Morente A, Oriol X, March-Llanes J. The Assertive Resolution of Conflicts in School With a Gamified Emotion Education Program. Frontiers in Psychology. 2018;9.

27. Allara E, Beccaria F, Molinar R, Marinaro L, Ermacora A, Coppo A, et al. A School-Based Program to Promote Well-Being in Preadolescents: Results From a Cluster Quasi-Experimental Controlled Study. Journal of Primary Prevention. 2019;40(2):151-70.

28. McMullen JD, McMullen N. Evaluation of a teacher-led, life-skills intervention for secondary school students in Uganda. Social Science & Medicine. 2018;217:10-7.

29. Fernández-Martínez I, Morales A, Espada JP, Essau CA, Orgilés M. Effectiveness of the program Super Skills For Life in reducing symptoms of anxiety and depression in young Spanish children. Psicothema. 2019;31(3):298-304.

30. Moulier V, Guinet H, Kovacevic Z, Bel-Abbass Z, Benamara Y, Zile N, et al. Effects of a life-skills-based prevention program on self-esteem and risk behaviors in adolescents: a pilot study. BMC Psychol. 2019;7(1):82.

31. Jafarigiv S, Peyman N. The effect of life skills training with health literacy strategies on self-esteem and self-efficacy in female students during puberty. International Journal of Adolescent Medicine and Health. 2019.

32. Lee M-J, Wu W-C, Chang H-C, Chen H-J, Lin W-S, Feng JY, et al. Effectiveness of a school-based life skills program on emotional regulation and depression among elementary school students: A randomized study. Children and Youth Services Review. 2020;118.

33. Heizomi H, Allahverdipour H, Jafarabadi MA, Bhalla D, Nadrian H. Effects of a mental health promotion intervention on mental health of Iranian female adolescents: a school-based study. Child and Adolescent Psychiatry and Mental Health. 2020;14(1).

34. Berger R, Benatov J, Cuadros R, VanNattan J, Gelkopf M. Enhancing resiliency and promoting prosocial behavior among Tanzanian primary-school students: A school-based intervention. Transcultural Psychiatry. 2018;55(6):821-45.

35. Khanna P, Singh K. Stress management training and gratitude journaling in the classroom: an initial investigation in Indian context. Current Psychology. 2021.

36. Fung J, Kim JJ, Jin J, Chen G, Bear L, Lau AS. A Randomized Trial Evaluating School-Based Mindfulness Intervention for Ethnic Minority Youth: Exploring Mediators and Moderators of Intervention Effects. Journal of Abnormal Child Psychology. 2019;47(1):1-19.

37. Devcich DA, Rix G, Bernay R, Graham E. Effectiveness of a Mindfulness-Based Program on School Children's Self-Reported Well-Being: A Pilot Study Comparing Effects With An Emotional Literacy Program. Journal of Applied School Psychology. 2017;33(4):309-30.

38. Sanger KL, Thierry G, Dorjee D. Effects of school-based mindfulness training on emotion processing and well-being in adolescents: evidence from event-related potentials. Dev Sci. 2018;21(5):e12646.

39. de Carvalho JS, Pinto AM, Maroco J. Results of a Mindfulness-Based Social-Emotional Learning Program on Portuguese Elementary Students and Teachers: a Quasi-Experimental Study. Mindfulness. 2017;8(2):337-50.

40. Kang Y, Rahrig H, Eichel K, Niles HF, Rocha T, Lepp NE, et al. Gender differences in response to a school-based mindfulness training intervention for early adolescents. J Sch Psychol. 2018;68:163-76.

41. Wright KM, Roberts R, Proeve MJ. Mindfulness-Based Cognitive Therapy for Children (MBCT-C) for Prevention of Internalizing Difficulties: a Small Randomized Controlled Trial with Australian Primary School Children. Mindfulness. 2019;10(11):2277-93.

42. Ghiroldi S, Scafuto F, Montecucco NF, Presaghi F, Iani L. Effectiveness of a School-Based Mindfulness Intervention on Children's Internalizing and Externalizing Problems: the Gaia Project. Mindfulness. 2020;11(11):2589-603.

43. Lam K, Seiden D. Effects of a Brief Mindfulness Curriculum on Self-reported Executive Functioning and Emotion Regulation in Hong Kong Adolescents. Mindfulness. 2020;11(3):627-42.

44. Lee RLT, Lane SJ, Tang ACY, Leung C, Louie LHT, Browne G, et al. Effects of an Unstructured Free Play and Mindfulness Intervention on Wellbeing in Kindergarten Students. Int J Environ Res Public Health. 2020;17(15).

45. Volanen SM, Lassander M, Hankonen N, Santalahti P, Hintsanen M, Simonsen N, et al. Healthy learning mind - Effectiveness of a mindfulness program on mental health compared to a relaxation program and teaching as usual in schools: A cluster-randomised controlled trial. J Affect Disord. 2020;260:660-9.

46. Amundsen R, Riby LM, Hamilton C, Hope M, McGann D. Mindfulness in primary school children as a route to enhanced life satisfaction, positive outlook and effective emotion regulation. BMC Psychol. 2020;8(1):71.

47. Johnson C, Burke C, Brinkman S, Wade T. A randomized controlled evaluation of a secondary school mindfulness program for early adolescents: Do we have the recipe right yet? Behav Res Ther. 2017;99:37-46.

48. Dray J, Bowman J, Campbell E, Freund M, Hodder R, Wolfenden L, et al. Effectiveness of a pragmatic school-based universal intervention targeting student resilience protective factors in reducing mental health problems in adolescents. Journal of Adolescence. 2017;57:74-89.

49. Kozina A. School-based prevention of anxiety using the "My FRIENDS" emotional resilience program: Six-month follow-up. International Journal of Psychology. 2020;55:70-7.

50. Olowokere AE, Okanlawon FA. Improving vulnerable school children's psychosocial health outcomes through resilience-based training and peer-support activities: a comparative prospective study. Vulnerable Children and Youth Studies. 2018;13(4):291-304.

51. Perry Y, Werner-Seidler A, Calear A, Mackinnon A, King C, Scott J, et al. Preventing Depression in Final Year Secondary Students: School-Based Randomized Controlled Trial. J Med Internet Res. 2017;19(11):e369.

52. Streimann K, Selart A, Trummal A. Effectiveness of a Universal, Classroom-Based Preventive Intervention (PAX GBG) in Estonia: a Cluster-Randomized Controlled Trial. Prev Sci. 2020;21(2):234-44.

53. Martinsen KD, Rasmussen LP, Wentzel-Larsen T, Holen S, Sund AM, Pedersen ML, et al. Change in quality of life and self-esteem in a randomized controlled CBT study for anxious and sad children: can targeting anxious and depressive symptoms improve functional domains in schoolchildren? BMC Psychol. 2021;9(1):8.

54. Garmy P, Clausson EK, Berg A, Steen Carlsson K, Jakobsson U. Evaluation of a school-based cognitive-behavioral depression prevention program. Scand J Public Health. 2019;47(2):182-9.

55. Redfern A, Jolley S, Bracegirdle K, Browning S, Plant D. Innovations in Practice: CUES-Ed: an in-service evaluation of a new universal cognitive behavioural early mental health intervention programme for primary school children. Child and Adolescent Mental Health. 2019;24(2):187-91.

56. Terry JD, Weist MD, Strait GG, Miller M. Motivational Interviewing to Promote the Effectiveness of Selective Prevention: an Integrated School-Based Approach. Prevention Science. 2020.

57. de la Torre-Luque A, Fiol-Veny A, Essau CA, Balle M, Bornas X. Effects of a transdiagnostic cognitive behaviour therapy-based programme on the natural course of anxiety symptoms in adolescence. J Affect Disord. 2020;264:474-82.

58. Lowe C, Wuthrich VM. Randomised Controlled Trial of Study Without Stress: A Cognitive Behavioural Therapy Program to Reduce Stress in Students in the Final Year of High School. Child Psychiatry & Human Development. 2021;52(2):205-16.

59. Klim-Conforti P, Zaheer R, Levitt AJ, Cheung AH, Schachar R, Schaffer A, et al. The Impact of a Harry Potter-Based Cognitive-Behavioral Therapy Skills Curriculum on Suicidality and Well-being in Middle Schoolers: A Randomized Controlled Trial. Journal of Affective Disorders. 2021;286:134-41.

60. Ahlen J, Lenhard F, Ghaderi A. Long-Term Outcome of a Cluster-Randomized Universal Prevention Trial Targeting Anxiety and Depression in School Children. Behav Ther. 2019;50(1):200-13.

61. Poppelaars M, Tak YR, Lichtwarck-Aschoff A, Engels RC, Lobel A, Merry SN, et al. A randomized controlled trial comparing two cognitive-behavioral programs for adolescent girls with subclinical depression: A school-based program (Op Volle Kracht) and a computerized program (SPARX). Behav Res Ther. 2016;80:33-42.

62. Waters AM, Candy SG, Zimmer-Gembeck MJ, Groth TA, Craske MG, Bradley BP, et al. A School-Based Comparison of Positive Search Training to Enhance Adaptive Attention Regulation with a Cognitive-Behavioural Intervention for Reducing Anxiety Symptoms in Children. J Abnorm Child Psychol. 2019;47(11):1821-40.

63. Bolling M, Niclasen J, Bentsen P, Nielsen G. Association of Education Outside the Classroom and Pupils' Psychosocial Well-Being: Results From a School Year Implementation. Journal of School Health. 2019;89(3):210-8.

64. Harvey DJ, Montgomery LN, Harvey H, Hall F, Gange AC, Watling D. Psychological benefits of a biodiversity-focussed outdoor learning program for primary school children. Journal of Environmental Psychology. 2020;67.

65. Ford T, Hayes R, Byford S, Edwards V, Fletcher M, Logan S, et al. The effectiveness and cost-effectiveness of the Incredible YearsA (R) Teacher Classroom Management programme in primary school children: results of the STARS cluster randomised controlled trial. Psychological Medicine. 2019;49(5):828-42.

66. Neal SC, Norwalk KE, Haskett ME. Differential impacts of the Incredible Years-Teacher Classroom Management program based on young children's risk profiles. Early Childhood Research Quarterly. 2020;51:473-82.

67. Benas JS, McCarthy AE, Haimm CA, Huang M, Gallop R, Young JF. The Depression Prevention Initiative: Impact on Adolescent Internalizing and Externalizing Symptoms in a Randomized Trial. J Clin Child Adolesc Psychol. 2019;48(sup1):S57-s71.

68. Van Ryzin MJ, Roseth CJ. The Cascading Effects of Reducing Student Stress: Cooperative Learning as a Means to Reduce Emotional Problems and Promote Academic Engagement. Journal of Early Adolescence. 2020.

69. Putwain DW, Pescod M. Is reducing uncertain control the key to successful test anxiety intervention for secondary school students? Findings from a randomized control trial. Sch Psychol Q. 2018;33(2):283-92.

70. Fergus TA, Limbers CA. Reducing Test Anxiety in School Settings: A Controlled Pilot Study Examining a Group Format Delivery of the Attention Training Technique Among Adolescent Students. Behav Ther. 2019;50(4):803-16.

71. Rizal H, Hajar MS, Muhamad AS, Kueh YC, Kuan G. The Effect of Brain Breaks on Physical Activity Behaviour among Primary School Children: A Transtheoretical Perspective. International Journal of Environmental Research and Public Health. 2019;16(21).

72. Cornelius C, Fedewa A, Toland M. A classroom-based physical activity intervention for adolescents: Is there an effect on self-efficacy, physical activity, and on-task behavior? Health Psychology Report. 2020;8(4):408-27.

73. Mok MMC, Chin M-K, Korcz A, Popeska B, Edginton CR, Uzunoz FS, et al. Brain Breaks (R) Physical Activity Solutions in the Classroom and on Attitudes toward Physical Activity: A Randomized Controlled Trial among Primary Students from Eight Countries. International Journal of Environmental Research and Public Health. 2020;17(5).

74. Sherry AP, Pearson N, Ridgers ND, Johnson W, Barber SE, Bingham DD, et al. Impacts of a Standing Desk Intervention within an English Primary School Classroom: A Pilot Controlled Trial. International Journal of Environmental Research and Public Health. 2020;17(19).

75. Costigan SA, Eather N, Plotnikoff RC, Hillman CH, Lubans DR. High-Intensity Interval Training for Cognitive and Mental Health in Adolescents. Med Sci Sports Exerc. 2016;48(10):1985-93.

76. Ruiz-Ariza A, Suarez-Manzano S, Lopez-Serrano S, Martinez-Lopez EJ. The effect of cooperative high-intensity interval training on creativity and emotional intelligence in secondary school: A randomised controlled trial. European Physical Education Review. 2019;25(2):355-73.

77. Cocca A, Espino Verdugo F, Ródenas Cuenca LT, Cocca M. Effect of a Game-Based Physical Education Program on Physical Fitness and Mental Health in Elementary School Children. Int J Environ Res Public Health. 2020;17(13).

78. Andrade A, Cruz WMD, Correia CK, Santos ALG, Bevilacqua GG. Effect of practice exergames on the mood states and self-esteem of elementary school boys and girls during physical education classes: A cluster-randomized controlled natural experiment. PLoS One. 2020;15(6):e0232392.

79. Zhang Y, Yin Y, Liu J, Yang M, Liu Z, Ma X. Impact of combined theory-based intervention on psychological effects and physical activity among chinese adolescents. International Journal of Environmental Research and Public Health. 2020;17(9).

80. Koszałka-Silska A, Korcz A, Wiza A. The impact of physical education based on the adventure education programme on self-esteem and social competences of adolescent boys. International Journal of Environmental Research and Public Health. 2021;18(6):1-14.

81. Resaland GK, Aadland E, Moe VF, Kolotkin RL, Anderssen SA, Andersen JR. Effects of a physical activity intervention on schoolchildren's health-related quality of life: The active smarter kids (ASK) cluster-randomized controlled trial. Preventive Medicine Reports. 2019;13:1-4.

82. Smith JJ, Beauchamp MR, Faulkner G, Morgan PJ, Kennedy SG, Lubans DR. Intervention effects and mediators of well-being in a school-based physical activity program for adolescents: The 'Resistance Training for Teens' cluster RCT. Mental Health and Physical Activity. 2018;15:88-94.

83. Luna P, Guerrero J, Cejudo J. Improving Adolescents' Subjective Well-Being, Trait Emotional Intelligence and Social Anxiety through a Programme Based on the Sport Education Model. Int J Environ Res Public Health. 2019;16(10).

84. Gall S, Walter C, du Randt R, Adams L, Joubert N, Mueller I, et al. Changes in Self-Reported Physical Activity Predict Health-Related Quality of Life Among South African Schoolchildren: Findings From the DASH Intervention Trial. Frontiers in Public Health. 2020;8.

85. Halliwell E, Jarman H, Tylka TL, Slater A. Evaluating the impact of a brief yoga intervention on preadolescents' body image and mood. Body Image. 2018;27:196-201.

86. Shreve M, Scott A, McNeill C, Washburn L. Using Yoga to Reduce Anxiety in Children: Exploring School-Based Yoga Among Rural Third- and Fourth-Grade Students. Journal of Pediatric Health Care. 2021;35(1):42-52.

87. Avitsland A, Leibinger E, Resaland GK, Solberg RB, Kolle E, Dyrstad SM. Effects of school-based physical activity interventions on mental health in adolescents: The School in Motion cluster randomized controlled trial. Mental Health and Physical Activity. 2020;19.

88. Schmidt SK, Reinboth MS, Resaland GK, Bratland-Sanda S. Changes in physical activity, physical fitness and well-being following a school-based health promotion program in a Norwegian region with a poor public health profile: A non-randomized controlled study in early adolescents. International Journal of Environmental Research and Public Health. 2020;17(3).

89. Annesi JJ, Walsh SM, Greenwood BL. Increasing Children's Voluntary Physical Activity Outside of School Hours Through Targeting Social Cognitive Theory Variables. J Prim Care Community Health. 2016;7(4):234-41.

90. Ho FKW, Louie LHT, Wong WH, Chan KL, Tiwari A, Chow CB, et al. A Sports-Based Youth Development Program, Teen Mental Health, and Physical Fitness: An RCT. Pediatrics. 2017;140(4).

91. Anusuya US, Mohanty S, Saoji AA. Effect of Mind Sound Resonance Technique (MSRT - A yoga-based relaxation technique) on psychological variables and cognition in school children: A randomized controlled trial. Complementary Therapies in Medicine. 2021;56.

92. McMahon K, Berger M, Khalsa KK, Harden E, Khalsa SBS. A Non-randomized Trial of Kundalini Yoga for Emotion Regulation within an After-school Program for Adolescents. Journal of Child and Family Studies. 2021;30(3):711-22.

93. Jayman M, Ohl M, Hughes B, Fox P. Improving socio-emotional health for pupils in early secondary education with Pyramid: A school-based, early intervention model. British Journal of Educational Psychology. 2019;89(1):111-30.

94. Duberg A, Jutengren G, Hagberg L, Möller M. The effects of a dance intervention on somatic symptoms and emotional distress in adolescent girls: A randomized controlled trial. J Int Med Res. 2020;48(2):300060520902610.

95. Kim H-S, Kim H-S. Effect of a musical instrument performance program on emotional intelligence, anxiety, and aggression in Korean elementary school children. Psychology of Music. 2018;46(3):440-53.

96. Shinde S, Weiss HA, Khandeparkar P, Pereira B, Sharma A, Gupta R, et al. A multicomponent secondary school health promotion intervention and adolescent health: An extension of the SEHER cluster randomised controlled trial in Bihar, India. PLoS Med. 2020;17(2):e1003021.

97. Shinde S, Weiss HA, Varghese B, Khandeparkar P, Pereira B, Sharma A, et al. Promoting school climate and health outcomes with the SEHER multi-component secondary school intervention in Bihar, India: a cluster-randomised controlled trial. Lancet. 2018;392(10163):2465-77.

98. Singla DR, Shinde S, Patton G, Patel V. The Mediating Effect of School Climate on Adolescent Mental Health: Findings From a Randomized Controlled Trial of a School-Wide Intervention. Journal of Adolescent Health. 2020.

99. Kiviruusu O, Björklund K, Koskinen HL, Liski A, Lindblom J, Kuoppamäki H, et al. Short-term effects of the "Together at School" intervention program on children's socio-emotional skills: a cluster randomized controlled trial. BMC Psychol. 2016;4(1):27.

100. Jansen M, Schroeders U, Luedtke O, Marsh HW. The dimensional structure of students' self-concept and interest in science depends on course composition. Learning and Instruction. 2019;60:20-8.

101. Nagamitsu S, Mimaki M, Koyanagi K, Tokita N, Kobayashi Y, Hattori R, et al. Prevalence and associated factors of suicidality in Japanese adolescents: Results from a population-based questionnaire survey. BMC Pediatrics. 2020;20(1).

102. Rodriguez S, Regueiro B, Pineiro I, Valle A, Sanchez B, Vieites T, et al. Success in Mathematics and Academic Wellbeing in Primary-School Students. Sustainability. 2020;12(9).

103. Holopainen L, Waltzer K, Hoang N, Lappalainen K. The Relationship between Students' Self-esteem, Schoolwork Difficulties and Subjective School Well-being in Finnish Upper-secondary Education. International Journal of Educational Research. 2020;104.

104. Yockey RA, King KA, Vidourek RA. School factors and anxiety disorder among Hispanic youth: Results from the 2016 US National Survey on Children's Health. School Psychology International. 2019;40(4):403-15.

105. Lonnfjord V, Hagquist C. The association of self-reported schoolwork pressure, family factors and self-efficacy with psychosomatic problems. European Journal of Social Work. 2020.

106. Yeo SC, Tan J, Lo JC, Chee MWL, Gooley JJ. Associations of time spent on homework or studying with nocturnal sleep behavior and depression symptoms in adolescents from Singapore. Sleep Health. 2020(2352-7226 (Electronic)).

107. Lv B, Lv L. Out-of-School Activities on Weekdays and Adolescent Adjustment in China: a Person-Centered Approach. Child Indicators Research. 2021;14(2):783-98.

108. Hamer M, Yates T, Sherar LB, Clemes SA, Shankar A. Association of after school sedentary behaviour in adolescence with mental wellbeing in adulthood. Prev Med. 2016;87:6-10.

109. Al-Qahtani AM, Al-Harbi MB. Prevalence and Risk Factors of Anxiety among Female Governmental Secondary Schools Students in Al-madinah, Saudi Arabia. Indian Journal of Pharmaceutical Education and Research. 2017;51(1):136-43.

110. Malak MZ, Khalifeh AH. Anxiety and depression among school students in Jordan: Prevalence, risk factors, and predictors. Perspect Psychiatr Care. 2018;54(2):242-50.

111. Sarı SA, Bilek G, Çelik E. Test anxiety and self-esteem in senior high school students: a cross-sectional study. Nord J Psychiatry. 2018;72(2):84-8.

112. Cikrikci O, Erzen E, Yeniceri IA. Self-Esteem and Optimism as Mediators in the Relationship Between Test Anxiety and Life Satisfaction Among a School-Based Sample of Adolescents. Journal of Psychologists and Counsellors in Schools. 2019;29(1):39-53.

113. Long E, Zucca C, Sweeting H. School Climate, Peer Relationships, and Adolescent Mental Health: A Social Ecological Perspective. Youth & Society. 2020.

114. Haerens L, Krijgsman C, Mouratidis A, Borghouts L, Cardon G, Aelterman N. How does knowledge about the criteria for an upcoming test relate to adolescents' situational motivation in physical education? A self-determination theory approach. European Physical Education Review. 2019;25(4):983-1001.

115. Nair S, Ganjiwale J, Kharod N, Varma J, Nimbalkar SM. Epidemiological survey of mental health in adolescent school children of Gujarat, India. BMJ Paediatrics Open. 2017;1(1).

116. Bashir MBA, Albadawy I, Cumber SN. Predictors and correlates of examination anxiety and depression among high school students taking the Sudanese national board examination in Khartoum state, Sudan: a cross-sectional study. Pan Afr Med J. 2019;33:69.

117. Evensen M. Adolescent Mental Health Problems, Behaviour Penalties, and Distributional Variation in Educational Achievement. European Sociological Review. 2019;35(4):474-90.

118. Torrano R, Ortigosa JM, Riquelme A, Mendez FJ, Lopez-Pina JA. Test Anxiety in Adolescent Students: Different Responses According to the Components of Anxiety as a Function of Sociodemographic and Academic Variables. Frontiers in Psychology. 2020;11.

119. Warne M, Snyder K, Gillander Gådin K. Participation and support - associations with Swedish pupils' positive health. Int J Circumpolar Health. 2017;76(1):1373579.

120. Oberle E. Early Adolescents' Emotional Well-Being in the Classroom: The Role of Personal and Contextual Assets. J Sch Health. 2018;88(2):101-11.

121. Povedano-Diaz A, Muñiz-Rivas M, Vera-Perea M. Adolescents' Life Satisfaction: The Role of Classroom, Family, Self-Concept and Gender. Int J Environ Res Public Health. 2019;17(1).

122. Yun JY, Chung H, Sim JA, Yun YH. Prevalence and associated factors of depression among Korean adolescents. PLoS One. 2019;14(10):e0223176.

123. Tong L, Reynolds K, Lee E, Liu Y. School Relational Climate, Social Identity, and Student Well-Being: New Evidence from China on Student Depression and Stress Levels. School Mental Health. 2019;11(3):509-21.

124. Herres J, Ewing ES, Kobak R. Emotional Reactivity to Negative Adult and Peer Events and the Maintenance of Adolescent Depressive Symptoms: a Daily Diary Design. J Abnorm Child Psychol. 2016;44(3):471-81.

125. Adewuya AO, Oladipo EO. Prevalence and associated factors for suicidal behaviours (ideation, planning, and attempt) among high school adolescents in Lagos, Nigeria. European Child & Adolescent Psychiatry. 2020;29(11):1503-12.

126. He GH, Strodl E, Chen WQ, Liu F, Hayixibayi A, Hou XY. Interpersonal Conflict, School Connectedness and Depressive Symptoms in Chinese Adolescents: Moderation Effect of Gender and Grade Level. Int J Environ Res Public Health. 2019;16(12).

127. Li J, Li J, Jia R, Wang Y, Qian S, Xu Y. Mental health problems and associated school interpersonal relationships among adolescents in China: a cross-sectional study. Child and Adolescent Psychiatry and Mental Health. 2020;14(1).

128. Fernandez-Sogorb A, Sanmartin R, Vicent M, Gonzalvez C. Identifying Profiles of Anxiety in Late Childhood and Exploring Their Relationship with School-Based Distress. International Journal of Environmental Research and Public Health. 2021;18(3).

129. Burns EC, Martin AJ, Collie RJ. Adaptability, personal best (PB) goals setting, and gains in students' academic outcomes: A longitudinal examination from a social cognitive perspective. Contemporary Educational Psychology. 2018;53:57-72.

130. Weyns T, Colpin H, Engels MC, Doumen S, Verschueren K. The relative contribution of peer acceptance and individual and class-level teacher-child interactions to kindergartners' behavioral development. Early Childhood Research Quarterly. 2019;47:259-70.

131. Pössel P, Burton SM, Cauley B, Sawyer MG, Spence SH, Sheffield J. Associations between Social Support from Family, Friends, and Teachers and depressive Symptoms in Adolescents. J Youth Adolesc. 2018;47(2):398-412.

132. Barnard AD, Adelson JL, Possel P. Associations between perceived teaching behaviours and affect in upper elementary school students. Early Child Development and Care. 2017;187(11):1795-808.

133. Cauley B, Possel P, Black SW, Hooper LM. Teaching Behavior and Positive and Negative Affect in High School Students: Does Students' Race Matter? School Mental Health. 2017;9(4):334-46.

134. Mizuta A, Suzuki K, Yamagata Z, Ojima T. Teachers' support and depression among Japanese adolescents: a multilevel analysis. Soc Psychiatry Psychiatr Epidemiol. 2017;52(2):211-9.

135. Quin D, Hemphill SA, Heerde JA. Associations between teaching quality and secondary students' behavioral, emotional, and cognitive engagement in school. Social Psychology of Education. 2017;20(4):807-29.

136. Guo C, Tomson G, Keller C, Söderqvist F. Prevalence and correlates of positive mental health in Chinese adolescents. BMC Public Health. 2018;18(1):263.

137. Bennefield Z. School and Family Correlates of Positive Affect in a Nationally Representative Sample of US Adolescents. Child and Adolescent Social Work Journal. 2018;35(5):541-8.

138. Corder K, Werneck AO, Jong ST, Hoare E, Brown HE, Foubister C, et al. Pathways to increasing adolescent physical activity and wellbeing: A mediation analysis of intervention components designed using a participatory approach. International Journal of Environmental Research and Public Health. 2020;17(2).

139. Lan X, Zhang L. Shields for Emotional Well-Being in Chinese Adolescents Who Switch Schools: The Role of Teacher Autonomy Support and Grit. Frontiers in Psychology. 2019;10.

140. Phan HP, Ngu BH. Schooling experience and academic performance of Taiwanese students: the importance of psychosocial effects, positive emotions, levels of best practice, and personal well-being. Social Psychology of Education. 2020;23(4):1073-101.

141. Pereyra SB, Bean RA, Ruiz JG, Velasco B. The Impact of Parents and Teachers on Externalizing Behavior Among Latino/a Adolescents via Academic Achievement: Combining the Mental Health and Educational Perspectives. Family Journal. 2020;28(3):290-9.

142. Kurdi V, Archambault I. Student-Teacher Relationships and Student Anxiety: Moderating Effects of Sex and Academic Achievement. Canadian Journal of School Psychology. 2018;33(3):212-26.

143. Shukla KD, Kuril S, Chand VS. Does negative teacher behavior influence student self-efficacy and mastery goal orientation? Learning and Motivation. 2020;71.

144. Wang C, Hatzigianni M, Shahaeian A, Murray E, Harrison LJ. The combined effects of teacher-child and peer relationships on children's social-emotional adjustment. J Sch Psychol. 2016;59:1-11.

145. Nie Q, Teng Z, Bear GG, Guo C, Liu Y, Zhang D. Hope as Mediator Between Teacher-Student Relationships and Life Satisfaction Among Chinese Adolescents: A Between- and Within-Person Effects Analysis. Journal of Happiness Studies. 2019;20(7):2367-83.

146. Wong MD, Dosanjh KK, Jackson NJ, Runger D, Dudovitz RN. The longitudinal relationship of school climate with adolescent social and emotional health. Bmc Public Health. 2021;21(1).

147. Xavier A, Cunha M, Pinto-Gouveia J. Daily Peer Hassles and Non-Suicidal Self-Injury in Adolescence: Gender Differences in Avoidance-Focused Emotion Regulation Processes. Journal of Child and Family Studies. 2018;27(1):59-68.

148. Lessard LM, Juvonen J. Friendless Adolescents: Do Perceptions of Social Threat Account for Their Internalizing Difficulties and Continued Friendlessness? J Res Adolesc. 2018;28(2):277-83.

149. Pandey AR, Bista B, Dhungana RR, Aryal KK, Chalise B, Dhimal M. Factors associated with suicidal ideation and suicidal attempts among adolescent students in Nepal: Findings from Global School-based Students Health Survey. PLoS One. 2019;14(4):e0210383.

150. Troop-Gordon W, MacDonald AP, Corbitt-Hall DJ. Children's Peer Beliefs, Friendlessness, and Friendship Quality: Reciprocal Influences and Contributions to Internalizing Symptoms. Developmental Psychology. 2019;55(11):2428-39.

151. Humenny G, Grygiel P, Dolata R, switaj P. Peer network Status and Depressive Symptoms Among Early Adolescents: Testing the Mediating Effects of Metaperception and Loneliness. School Mental Health. 2021.

152. Biswas T, Scott JG, Munir K, Renzaho AMN, Rawal LB, Baxter J, et al. Global variation in the prevalence of suicidal ideation, anxiety and their correlates among adolescents: A population based study of 82 countries. EClinicalMedicine. 2020;24.

153. Schmidt A, Neubauer AB, Dirk J, Schmiedek F. The Bright and the Dark Side of Peer Relationships: Differential Effects of Relatedness Satisfaction and Frustration at School on Affective Well-Being in Children's Daily Lives. Developmental Psychology. 2020;56(8):1532-46.

154. Carter R, Leath S, Butler-Barnes ST, Bryd CM, Chavous TM, Caldwell CH, et al. Comparing Associations Between Perceived Puberty, Same-Race Friends and Same-Race Peers, and Psychosocial Outcomes Among African American and Caribbean Black Girls. Journal of Black Psychology. 2017;43(8):836-62.

155. Attar-Schwartz S, Mishna F, Khoury-Kassabri M. The Role of Classmates' Social Support, Peer Victimization and Gender in Externalizing and Internalizing Behaviors among Canadian Youth. Journal of Child and Family Studies. 2019;28(9):2335-46.

156. Baytemir K. Experiences of School as a Mediator between Interpersonal Competence and Happiness in Adolescents. Anales De Psicologia. 2019;35(2):259-68.

157. Wu N, Hou Y, Chen P, You J. Peer Acceptance and Nonsuicidal Self-injury among Chinese Adolescents: A Longitudinal Moderated Mediation Model. J Youth Adolesc. 2019;48(9):1806-17.

158. Copeland M, Siennick SE, Feinberg ME, Moody J, Ragan DT. Social Ties Cut Both Ways: Self-Harm and Adolescent Peer Networks. J Youth Adolesc. 2019;48(8):1506-18.

159. Dang J, Liu L, Du Y. Benefits of a Highly Entitative Class for Adolescents' Psychological Well-Being in School. School Mental Health. 2019;11(4):766-76.

160. Lyell KM, Coyle S, Malecki CK, Santuzzi AM. Parent and peer social support compensation and internalizing problems in adolescence. Journal of School Psychology. 2020;83:25-49.

161. Fussner LM, Luebbe AM, Mancini KJ, Becker SP. Emotion dysregulation mediates the longitudinal relation between peer rejection and depression: Differential effects of gender and grade. International Journal of Behavioral Development. 2018;42(2):155-66.

162. Zhang S, Baams L, van de Bongardt D, Dubas JS. Intra- and Inter-Individual Differences in Adolescent Depressive Mood: the Role of Relationships with Parents and Friends. J Abnorm Child Psychol. 2018;46(4):811-24.

163. Mali LV, Schwartz D, Badaly D, Luo TJ, Malamut S, Ross AC, et al. Unpopularity with same- and cross-ethnicity peers as predictors of depressive symptoms during adolescence. Journal of Applied Developmental Psychology. 2019;62:93-101.

164. Martinez G, Bámaca-Colbert MY. A Reciprocal and Longitudinal Investigation of Peer and School Stressors and Depressive Symptoms Among Mexican-Origin Adolescent Females. J Youth Adolesc. 2019;48(11):2125-40.

165. Delgado MY, Nair RL, Updegraff KA, Umaña-Taylor AJ. Discrimination, Parent-Adolescent Conflict, and Peer Intimacy: Examining Risk and Resilience in Mexican-Origin Youths' Adjustment Trajectories. Child Dev. 2019;90(3):894-910.

166. Laetsch A. Do perceived helpfulness and competition in classroom contexts moderate or mediate the association between perceived stress and socio-emotional strengths and difficulties from early to middle adolescence? Learning and Individual Differences. 2017;58:31-40.

167. Wood MA, Bukowski WM, Santo JB. Friendship Security, But Not Friendship Intimacy, Moderates the Stability of Anxiety During Preadolescence. J Clin Child Adolesc Psychol. 2017;46(6):798-809.

168. van Harmelen AL, Gibson JL, St Clair MC, Owens M, Brodbeck J, Dunn V, et al. Friendships and Family Support Reduce Subsequent Depressive Symptoms in At-Risk Adolescents. PLoS One. 2016;11(5):e0153715.

169. King RB, Datu JA. Happy classes make happy students: Classmates' well-being predicts individual student well-being. J Sch Psychol. 2017;65:116-28.

170. Ng-Knight T, Shelton KH, Riglin L, Frederickson N, McManus IC, Rice F. 'Best friends forever'? Friendship stability across school transition and associations with mental health and educational attainment. British Journal of Educational Psychology. 2019;89(4):585-99.

171. Oberle E, Guhn M, Gadermann AM, Thomson K, Schonert-Reichl KA. Positive mental health and supportive school environments: A population-level longitudinal study of dispositional optimism and school relationships in early adolescence. Soc Sci Med. 2018;214:154-61.

172. Tetzner J, Becker M. Why are you so optimistic? Effects of sociodemographic factors, individual experiences, and peer characteristics on optimism in early adolescents. J Pers. 2019;87(3):661-75.

173. Chen Y, Wang L, Zhao J. Peer relationship profiles in rural Chinese adolescents: Longitudinal relations with subjective well-being. Journal of Health Psychology. 2019.

174. Yang Y, Chen L, Zhang L, Ji L, Zhang W. Developmental changes in associations between depressive symptoms and peer relationships: a four-year follow-up of Chinese adolescents. J Youth Adolesc. 2020;49(9):1913-27.

175. Farren GL, Zhang T, Gu X, Thomas KT. Sedentary behavior and physical activity predicting depressive symptoms in adolescents beyond attributes of health-related physical fitness. Journal of Sport and Health Science. 2018;7(4):489-96.

176. Belton S, Issartel J, McGrane B, Powell D, O'Brien W. A consideration for physical literacy in Irish youth, and implications for physical education in a changing landscape. Irish Educational Studies. 2019;38(2):193-211.

177. Barth Vedøy I, Anderssen SA, Tjomsland HE, Skulberg KR, Thurston M. Physical activity, mental health and academic achievement: A cross-sectional study of Norwegian adolescents. Mental Health and Physical Activity. 2020;18.

178. Wunsch K, Nigg CR, Weyland S, Jekauc D, Niessner C, Burchartz A, et al. The relationship of self-reported and device-based measures of physical activity and health-related quality of life in adolescents. Health and Quality of Life Outcomes. 2021;19(1).

179. Park S, Park SY, Jang SY, Oh G, Oh IH. The Neglected Role of Physical Education Participation on Suicidal Ideation and Stress in High School Adolescents from South Korea. Int J Environ Res Public Health. 2020;17(8).

180. Bell SL, Audrey S, Gunnell D, Cooper A, Campbell R. The relationship between physical activity, mental wellbeing and symptoms of mental health disorder in adolescents: A cohort study. International Journal of Behavioral Nutrition and Physical Activity. 2019;16(1).

181. Opdal IM, Morseth B, Handegård BH, Lillevoll K, Ask H, Nielsen CS, et al. Change in physical activity is not associated with change in mental distress among adolescents: the Tromsø study: Fit Futures. BMC Public Health. 2019;19(1):916.

182. Hyakutake A, Kamijo T, Misawa Y, Washizuka S, Inaba Y, Tsukahara T, et al. Cross-sectional observation of the relationship of depressive symptoms with lifestyles and parents' status among Japanese junior high school students. Environ Health Prev Med. 2016;21(4):265-73.

183. McMahon EM, Corcoran P, O'Regan G, Keeley H, Cannon M, Carli V, et al. Physical activity in European adolescents and associations with anxiety, depression and well-being. Eur Child Adolesc Psychiatry. 2017;26(1):111-22.

184. Tajik E, Abd Latiff L, Adznam SN, Awang H, Yit Siew C, Abu Bakar AS. A study on level of physical activity, depression, anxiety and stress symptoms among adolescents. J Sports Med Phys Fitness. 2017;57(10):1382-7.

185. Kleppang AL, Thurston M, Hartz I, Hagquist C. Psychological distress among Norwegian adolescents: Changes between 2001 and 2009 and associations with leisure time physical activity and screen-based sedentary behaviour. Scand J Public Health. 2019;47(2):166-73.

186. Bélair MA, Kohen DE, Kingsbury M, Colman I. Relationship between leisure time physical activity, sedentary behaviour and symptoms of depression and anxiety: evidence from a population-based sample of Canadian adolescents. BMJ Open. 2018;8(10):e021119.

187. O'Brien K, Agostino J, Ciszek K, Douglas KA. Physical activity and risk of behavioural and mental health disorders in kindergarten children: analysis of a series of cross-sectional complete enumeration (census) surveys. BMJ Open. 2020;10(3):e034847.

188. Guddal MH, Stensland S, Småstuen MC, Johnsen MB, Zwart JA, Storheim K. Physical activity and sport participation among adolescents: associations with mental health in different age groups. Results from the Young-HUNT study: a cross-sectional survey. BMJ Open. 2019;9(9):e028555.

189. Oosterhoff B, Kaplow JB, Wray-Lake L, Gallagher K. Activity-specific pathways among duration of organized activity involvement, social support, and adolescent well-being: Findings from a nationally representative sample. J Adolesc. 2017;60:83-93.

190. Reverdito RS, Carvalho HM, Galatti LR, Scaglia AJ, Gonçalves CE, Paes RR. Effects of Youth Participation in Extra-Curricular Sport Programs on Perceived Self-Efficacy: A Multilevel Analysis. Percept Mot Skills. 2017;124(3):569-83.

191. Kleppang AL, Hartz I, Thurston M, Hagquist C. The association between physical activity and symptoms of depression in different contexts - a cross-sectional study of Norwegian adolescents. BMC Public Health. 2018;18(1):1368.

192. Oberle E, Ji XR, Kerai S, Guhn M, Schonert-Reichl KA, Gadermann AM. Screen time and extracurricular activities as risk and protective factors for mental health in adolescence: A population-level study. Preventive Medicine. 2020;141.

193. Jiang R, Xie C, Shi J, Mao X, Huang Q, Meng F, et al. Comparison of physical fitness and mental health status among school-age children with different sport-specific training frequencies. Peerj. 2021;9.

194. Guzmán-Rocha MD, McLeod DL, Bohnert AM. Dimensions of organized activity involvement among Latino youth: Impact on well-being. J Adolesc. 2017;60:130-9.

195. Moeijes J, van Busschbach JT, Bosscher RJ, Twisk JWR. Sports participation and psychosocial health: a longitudinal observational study in children. BMC Public Health. 2018;18(1):702.

196. Oberle E, Ji XR, Guhn M, Schonert-Reichl KA, Gadermann AM. Benefits of Extracurricular Participation in Early Adolescence: Associations with Peer Belonging and Mental Health. J Youth Adolesc. 2019;48(11):2255-70.

197. Tu HM. Does active leisure participation promote psychological capital through peer support in economically disadvantaged children? PLoS One. 2020;15(6):e0234143.
